# The Subtype of GluN2 C-terminal Domain Determines the Response to Excitotoxic Insults

**DOI:** 10.1016/j.neuron.2012.03.021

**Published:** 2012-05-10

**Authors:** Marc-André Martel, Tomás J. Ryan, Karen F.S. Bell, Jill H. Fowler, Aoife McMahon, Bashayer Al-Mubarak, Noboru H. Komiyama, Karen Horsburgh, Peter C. Kind, Seth G.N. Grant, David J.A. Wyllie, Giles E. Hardingham

**Affiliations:** 1 Centre for Integrative Physiology, University of Edinburgh School of Biomedical Sciences, Hugh Robson Building, George Square, Edinburgh EH8 9XD, UK; 2 Wellcome Trust Sanger Institute, Hinxton CB10 1SA, UK; 3 Wolfson College, University of Cambridge, Barton Road, Cambridge CB3 9BB, UK; 4 Centre for Neuroregeneration, University of Edinburgh Chancellor's Building, Edinburgh EH16 4SB, UK; 5 Centre for Clinical Brain Sciences and Centre for Neuroregeneration, University of Edinburgh Chancellor's Building, Edinburgh, EH16 4SB, UK

## Abstract

It is currently unclear whether the GluN2 subtype influences NMDA receptor (NMDAR) excitotoxicity. We report that the toxicity of NMDAR-mediated Ca^2+^ influx is differentially controlled by the cytoplasmic C-terminal domains of GluN2B (CTD^2B^) and GluN2A (CTD^2A^). Studying the effects of acute expression of GluN2A/2B-based chimeric subunits with reciprocal exchanges of their CTDs revealed that CTD^2B^ enhances NMDAR toxicity, compared to CTD^2A^. Furthermore, the vulnerability of forebrain neurons in vitro and in vivo to NMDAR-dependent Ca^2+^ influx is lowered by replacing the CTD of *GluN2B* with that of *GluN2A* by targeted exon exchange in a mouse knockin model. Mechanistically, CTD^2B^ exhibits stronger physical/functional coupling to the PSD-95-nNOS pathway, which suppresses protective CREB activation. Dependence of NMDAR excitotoxicity on the GluN2 CTD subtype can be overcome by inducing high levels of NMDAR activity. Thus, the identity (2A versus 2B) of the GluN2 CTD controls the toxicity dose-response to episodes of NMDAR activity.

## Highlights

► The CTD of GluN2B promotes excitotoxicity better than that of GluN2A ► GluN2 CTD subtype differences are seen in both WT and chimeric 2A/2B subunits ► The GluN2B CTD couples to a prodeath PSD-95/nNOS-dependent CREB shut-off pathway

## Teaser

Martel et al. find that the two subtypes (2A versus 2B) of the GluN2 C-terminal domain differentially couple to the CREB shut-off pathway, causing distinct effects on NMDA receptor-mediated neuronal death both in vitro and in vivo.

## Introduction

Sustained elevated levels of extracellular glutamate kill central neurons (Olney, 1969). This “excitotoxicity” is implicated in neuronal loss in acute neurological disorders, including stroke, traumatic brain injury, and chronic disorders including Huntington's disease (Berliocchi et al., 2005; Choi, 1988; Fan and Raymond, 2007; Lau and Tymianski, 2010). A major cause of glutamate excitotoxicity is inappropriate activity of the NMDA subtype of glutamate receptor (NMDAR), which mediates Ca^2+^-dependent cell death (Choi, 1992; Lipton, 2006). Most NMDARs contain two obligate GluN1 subunits plus two GluN2 subunits (Furukawa et al., 2005), of which there are four subtypes, GluN2A-D, with GluN2A and GluN2B predominant in the forebrain (Cull-Candy et al., 2001; Monyer et al., 1994; Paoletti, 2011; Traynelis et al., 2010). GluN2 subunits have large, evolutionarily divergent cytoplasmic C-terminal domains (CTDs), which have the potential to differentially associate with signaling molecules (Ryan et al., 2008). This compositional diversity raises the (unresolved) question as to whether the GluN2 subtype (GluN2A versus GluN2B) differentially influences the toxicity of Ca^2+^ influx through NMDARs. There is evidence that GluN2B- and GluN2A-containing NMDARs are both capable of mediating excitotoxicity (Graham et al., 1992; Lau and Tymianski, 2010; von Engelhardt et al., 2007); however, whether they do so with differing efficiency or mechanisms is unclear.

In answering questions relating to subunit-specific function (including excitotoxicity), it is becoming clear that pharmacological approaches are of limited use, given the tools currently available (Neyton and Paoletti, 2006). Although GluN2B-specific antagonists are highly selective and have demonstrated a role for GluN2B-containing NMDARs in excitotoxicity (Liu et al., 2007), attempts to study the role of GluN2A (Liu et al., 2007) employed a mildly selective GluN2A-preferring antagonist (NVP-AAM007) at a concentration shown by others to antagonize GluN2B-containing NMDARs (Berberich et al., 2005; Frizelle et al., 2006; Martel et al., 2009; Neyton and Paoletti, 2006; Weitlauf et al., 2005), rendering some of the findings hard to interpret. Moreover, the less-controllable conditions in an intact brain render a weakly selective competitive antagonist, such as NVP-AAM007, of limited value for in vivo studies. Another important issue is that receptors can exist in a triheteromeric form that contains both a GluN2A and a GluN2B subunit (Hatton and Paoletti, 2005; Rauner and Köhr, 2011), where the role of each subunit cannot be established using currently available pharmacological tools.

Additional problems in relating function to GluN2 subunit composition include their different spatiotemporal expression profiles. For example, in younger neurons, GluN2B is predominant and as such may mediate excitotoxicity simply because most NMDARs are GluN2B-containing. Moreover, GluN2B- and GluN2A-containing NMDARs may be enriched at extrasynaptic and synaptic sites, respectively (Groc et al., 2006; Martel et al., 2009; Tovar and Westbrook, 1999, but see Harris and Pettit, 2007; Thomas et al., 2006). Since receptor location may be a determinant of excitotoxicity irrespective of subunit composition (Hardingham and Bading, 2010), a location-dependent effect may be misinterpreted as a subunit-specific effect.

We have eschewed pharmacocentric approaches in favor of molecular genetics to determine whether equivalent levels of Ca^2+^ influx through GluN2A- and GluN2B-containing NMDARs differentially affect neuronal viability. We hypothesized that any differences would be due to their large CTDs because this is the primary area of sequence divergence, as well as being the part of GluN2 known to bind intracellular signaling/scaffolding proteins (Ryan et al., 2008). By studying signaling from wild-type and chimeric GluN2A/2B subunits, using both acutely expressed subunits as well as a mouse knockin model, we find that the presence of the CTD^2B^ in an NMDAR renders Ca^2+^ influx through this receptor more toxic than the presence of CTD^2A^. This difference is observed in vivo as well as in vitro and is attributable in part to enhanced physical/functional coupling of CTD^2B^ to the PSD-95/nNOS signaling cassette, which suppresses prosurvival CREB-mediated gene expression, rendering neurons vulnerable to excitotoxic cell death.

## Results

### The CTDs of GluN2B and GluN2A Differentially Influence Excitotoxicity Independent of the Identity of the Rest of the Subunit

**Figure 1:**
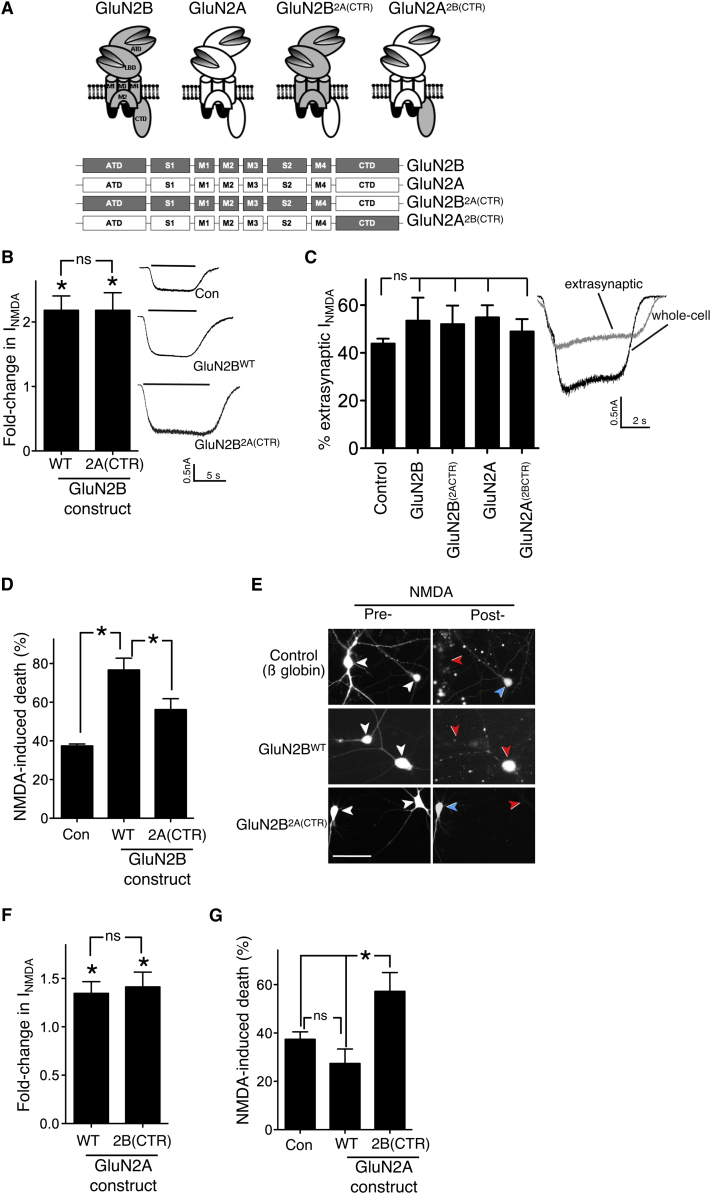
The GluN2B C-Terminal Domain Promotes NMDAR-Mediated Toxicity When Linked to Either Channel Portion of GluN2B or GluN2A (A) Schematic and linear representation of GluN2A, GluN2B, and the chimeric subunits in which the C-terminal domain (CTD) has been replaced (CTR). Constructs encoding these subunits were expressed in hippocampal neurons. ATD, amino-terminal domain; S1-S2, extracellular ligand-binding domains (LBD); M1-M4, intramembranous domains. (B) Acute expression of GluN2B^WT^ or GluN2B^2A(CTR)^ has a similar effect on NMDA-induced whole-cell currents. Neurons were transfected with the indicated constructs (plus eGFP marker) and whole-cell steady-state NMDAR-mediated currents evoked by 20 μM NMDA (and normalized to cell capacitance, here and throughout) were compared to control-transfected neurons (β-globin, n = 12–14 cells per construct) ^∗^ p < 0.05 (t test comparison to control-transfected neurons). Responses, here and throughout, were measured at 48 hr posttransfection. Mean ± SEM shown here and throughout the figure. (C) Expression of the subunits described in (A) does not alter the overall proportion of extrasynaptic NMDARs (n = 5–10 cells for each construct). Right shows example trace of NMDAR-mediated currents before (whole cell) and after synaptic NMDAR blockade (extrasynaptic). See Supplemental Experimental Procedures for details. (D) GluN2B^WT^ expression renders neurons more vulnerable to an excitotoxic insult (20 μM NMDA for 1 hr), but replacing the CTD to that of GluN2A reduces the level of toxicity (^∗^p < 0.05; n = 7; 150–200 cells analyzed per condition). (E) Example pictures of (D) showing transfected cells with the relevant plasmid (+eGFP) pre- and post-NMDA treatment. White arrows indicate transfected neurons before NMDA treatment. Red/blue arrows in the “posttreatment” panels indicate dead/live cells, respectively. (F) Expression of GluN2A^WT^ or GluN2A^2B(CTR)^ enhances NMDAR currents to similar levels compared to globin-expressing cells (n = 10–11 cells per construct). ^∗^p < 0.05 (t test comparison to control-transfected neurons). (G) NMDA-induced toxicity is significantly higher in GluN2A^2B(CTR)^-transfected neurons than with GluN2A^WT^ (^∗^p < 0.05; n = 8). See also Figure S1.

We wanted to investigate whether the subtype of GluN2 CTD influences the excitotoxicity of a given amount of NMDAR-mediated ion flux. We created constructs encoding chimeric receptors based on GluN2B and GluN2A but with their respective CTDs replaced (denoted as CTR) with each other's (GluN2B^2A(CTR)^ and GluN2A^2B(CTR)^, respectively, Figure 1A). In rat hippocampal neurons, we first expressed either wild-type GluN2B^WT^ or GluN2B^2A(CTR)^, at a developmental stage where endogenous NMDARs are overwhelmingly GluN2B-containing (Martel et al., 2009). Expression of GluN2B^WT^ or GluN2B^2A(CTR)^ both enhanced whole-cell currents to a similar level (Figure 1B) and did not differentially affect the proportion of extrasynaptic NMDARs (Figure 1C), as assessed by the “quantal block” method of irreversibly blocking synaptically located NMDARs (Papadia et al., 2008). Thus, any differential CTD-specific effects on excitotoxicity can be studied without the complicating factor of altered NMDAR location, which itself influences survival/death signaling via mechanisms that are likely to be independent of GluN2 subtype (Hardingham and Bading, 2010; Martel et al., 2009; Papadia et al., 2008).

We next studied whether expression of GluN2B^WT^ or GluN2B^2A(CTR)^ had different effects on vulnerability to excitotoxicity. NMDA (20 μM) was applied for 1 hr to neurons transfected with vectors encoding either GluN2B^WT^, GluN2B^2A(CTR)^ or control vector, and neuronal death was assessed 24 hr later. GluN2B^WT^ strongly increased the level of cell death compared to the control, consistent with NMDAR currents being higher (Figures 1D and 1E). However, expression of GluN2B^2A(CTR)^ caused a significantly lower enhancement of cell death than GluN2B^WT^ (Figures 1D and 1E), despite NMDAR currents being equal (Figure 1B), suggesting that CTD^2B^ promotes cell death better than CTD^2A^. The same result was found when the experiment was repeated in DIV18 neurons (see Figure S1A available online), indicating that the differential effect of CTD^2B^ versus CTD^2A^ on cell death also holds true in more mature neurons.

To further investigate the differential CTD subtype effects on excitotoxicity, we compared NMDAR-dependent cell death in neurons expressing GluN2A^WT^ and GluN2A^2B(CTR)^. Expression of GluN2A^WT^ and GluN2A^2B(CTR)^ did not differentially affect the proportion of extrasynaptic NMDARs (Figure 1C) and caused similar increases in NMDAR currents (Figure 1F); although, because of the lower affinity of GluN2A for NMDA, the increases were smaller than for the GluN2B-based constructs (Figure 1B). We found that neurons expressing GluN2A^2B(CTR)^ were significantly more vulnerable to NMDA-induced excitotoxicity than GluN2A^WT^-expressing neurons (Figure 1G). Thus, for a given amount of NMDAR-mediated current, the presence of CTD^2B^ promotes neuronal death better than CTD^2A^, regardless of whether they are linked to the channel portion of GluN2A or GluN2B. This result illustrates the independent influence of the identity of the CTD on excitotoxicity, acting in addition to the influence of the identity of the rest of the channel on downstream signaling events (e.g., because of different channel kinetics and ligand binding properties).

### A Mouse Knockin Model Reveals the Influence of the GluN2 CTD Subtype In Vitro and In Vivo

**Figure 2:**
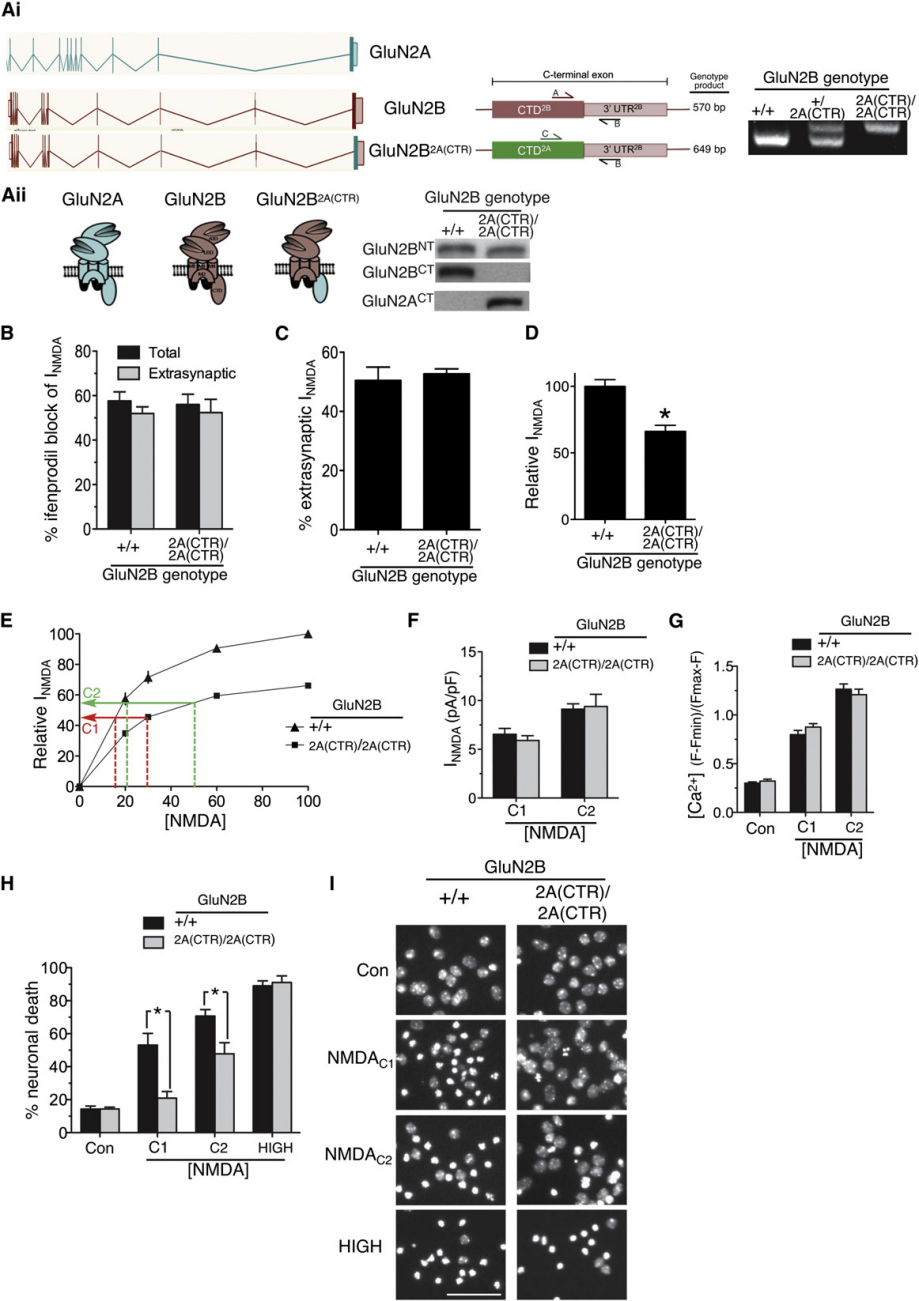
Replacement of the GluN2B C-Terminal Domain with that of GluN2A in a Mouse Knockin Model Decreases NMDAR-Mediated Excitotoxicity in Mouse Cortical Neurons (Ai) (Left) Linear representations of the *GluN2A* and *GluN2B* genes and of the knockin mouse line *GluN2B*^2A(CTR)^, in which the protein coding region of the C-terminal exon of *GluN2B* (867G to 1482V) was replaced with that of *GluN2A* (866G to 1464V). (Middle) Schematic focusing on the C-terminal exon of GluN2B, illustrating the location of the genotyping primers. Note that a common reverse primer (primer “B” within the GluN2B 3′ UTR) is used for both reactions, together with a forward primer specific for either the GluN2B (primer “A”) or GluN2A CTD (primer “C”). (Right) Example of genotyping products obtained in wild-type, heterozygotes, and homozygous knockin mice. (Aii) (Left) Cartoon illustrating the gene products of *GluN2A*, *GluN2B^WT^*, and *GluN2B*^2A(CTR)^ (green = GluN2A; red = GluN2B). (Right) Western blot of protein extracts obtained from *GluN2B*^+/+^ and *GluN2B*^2A(CTR)/2A(CTR)^ cortical neurons at DIV8 (when levels of GluN2A are extremely low). Note that, whereas the N-terminal antibody picks up both GluN2B^WT^ and GluN2B^2A(CTR)^, an antibody specific for the CTD of GluN2B only picks up GluN2B^WT^, and an antibody specific for the CTD of GluN2A picks up GluN2B^2A(CTR)^. (B) The effect of ifenprodil (3 μM) on total and extrasynaptic NMDAR currents was measured in *GluN2B*^+/+^ and *GluN2B*^2A(CTR)/2A(CTR)^ DIV10 cortical neurons (n = 9 cells per genotype [total]; n = 4 per genotype [extrasynaptic]). NMDAR currents were measured at the steady state and normalized to cell capacitance (here and throughout). Mean ± SEM shown here and throughout the figure. (C) The proportion of steady-state extrasynaptic NMDAR currents as a percentage of whole-cell currents was analyzed in *GluN2B*^+/+^ and *GluN2B*^2A(CTR)/2A(CTR)^ (see Experimental Procedures; n = 8). (D) Whole-cell NMDAR responses (evoked by 100 μM NMDA) are lower in *GluN2B*^2A(CTR)/2A(CTR^ neurons (n = 33) compared to *GluN2B*^+/+^ (n = 43). Steady-state NMDAR currents in *GluN2B*^2A(CTR)/2A(CTR^ neurons were expressed as a percentage of those obtained in *GluN2B*^+/+^ neurons. (E) Calculation of NMDA concentrations (C1 and C2) predicted to trigger equivalent NMDAR currents in *GluN2B*^+/+^ and *GluN2B*^2A(CTR)/2A(CTR)^ neurons, based on dose response curves (n = 8 cells for each curve). Relative NMDAR currents are expressed as a percentage of the maximum current obtained in *GluN2B*^+/+^ neurons. (F–G) NMDA_C1_ and NMDA_C2_ both evoke similar NMDAR currents and increases in free Ca^2+^ in *GluN2B*^+/+^ and *GluN2B*^2A(CTR)/2A(CTR)^ neurons. (F) NMDAR currents were measured (n = 7–8 cells per condition) and (G) Fluo-3 Ca^2+^ imaging was performed where between 90 and 105 cells were analyzed within 3 independent experiments. (H) NMDA-induced cell death is diminished in neurons containing *GluN2B*^2A(CTR)^ compared to *GluN2B*^WT^. *GluN2B*^+/+^ and *GluN2B*^2A(CTR)/2A(CTR)^ neurons were treated for 1 hr with NMDA_C1_, NMDA_C2_, or a high (100 μM) dose of NMDA. Cell death was analyzed after 24 hr (^∗^p < 0.05; n = 11 (*GluN2B*^+/+^); n = 15 (*GluN2B*^2A(CTR)/2A(CTR)^; 13,000–21,000 cells analyzed per treatment per genotype). (I) Example pictures from (H). Scale bar 50 μm. See also Figure S2.

We next investigated the importance of the GluN2 CTD subtype by an independent approach: a genetically modified “knockin” mouse in which the protein coding portion of the C-terminal exon of *GluN2B* (encoding over 95% of the CTD) was exchanged for that of *GluN2A* (*GluN2B*^2A(CTR)^; Figure 2A; see Supplemental Experimental Procedures). The 3′UTR of GluN2B, which also forms part of the C-terminal exon, was unchanged apart from a 61 bp insertion at its beginning (a remnant of the excision of a neomycin resistance selection cassette). We wanted to determine whether equivalent Ca^2+^ influx through GluN2B-containing and GluN2B^2A(CTR)^-containing NMDARs would result in different levels of neuronal death. We studied DIV10 cultured cortical neurons from *GluN2B*^+/+^ and *GluN2B*^2A(CTR)/2A(CTR)^ littermates. These cultures exhibited similar levels of basal viability and levels of synaptic connectivity and strength, as measured by mini EPSC frequency/size, spontaneous EPSC frequency, and AMPA receptor currents (Figures S2A–S2D), as well as unaltered cell capacitance (Figure S2E).

Whole-cell and extrasynaptic NMDAR currents in both *GluN2B*^+/+^ and *GluN2B*^2A(CTR)/2A(CTR)^ neurons were found to be similarly sensitive to the GluN2B-specific antagonist ifenprodil. In neurons of both genotypes, we observed a blockade of around 60% (Figure 2B), indicative of a high (∼80%) level of GluN1/GluN2B heterodimeric receptors. Moreover, the proportion of extrasynaptic NMDARs was found to be the same for *GluN2B*^2A(CTR)/2A(CTR)^ and *GluN2B*^+/+^ neurons (Figure 2C). Thus, any differential CTD subtype-specific effects on excitotoxicity could be studied without the potentially confounding factor of altered NMDAR location. We also investigated whether any differences in use-dependent run-down of whole-cell NMDAR currents were observed because this may be relevant to long-term exposure to NMDA. Having measured baseline whole-cell NMDAR currents, ten further 10 s applications of NMDA were applied over a 10 min period. We found no difference in run-down of steady-state NMDAR currents in *GluN2B*^+/+^ and *GluN2B*^2A(CTR)/2A(CTR)^ neurons (around 3% per application; Figure S2F). We also examined NMDAR single-channel properties. We excised outside-out patches from DIV9 *GluN2B*^+/+^ and *GluN2B*^2A(CTR)/2A(CTR)^ neurons and measured NMDA-evoked unitary currents, finding no difference in their mean single-channel conductance of approximately 50 pS, which is typical for GluN2B-containing NMDARs (Figure S2G).

**Figure 3:**
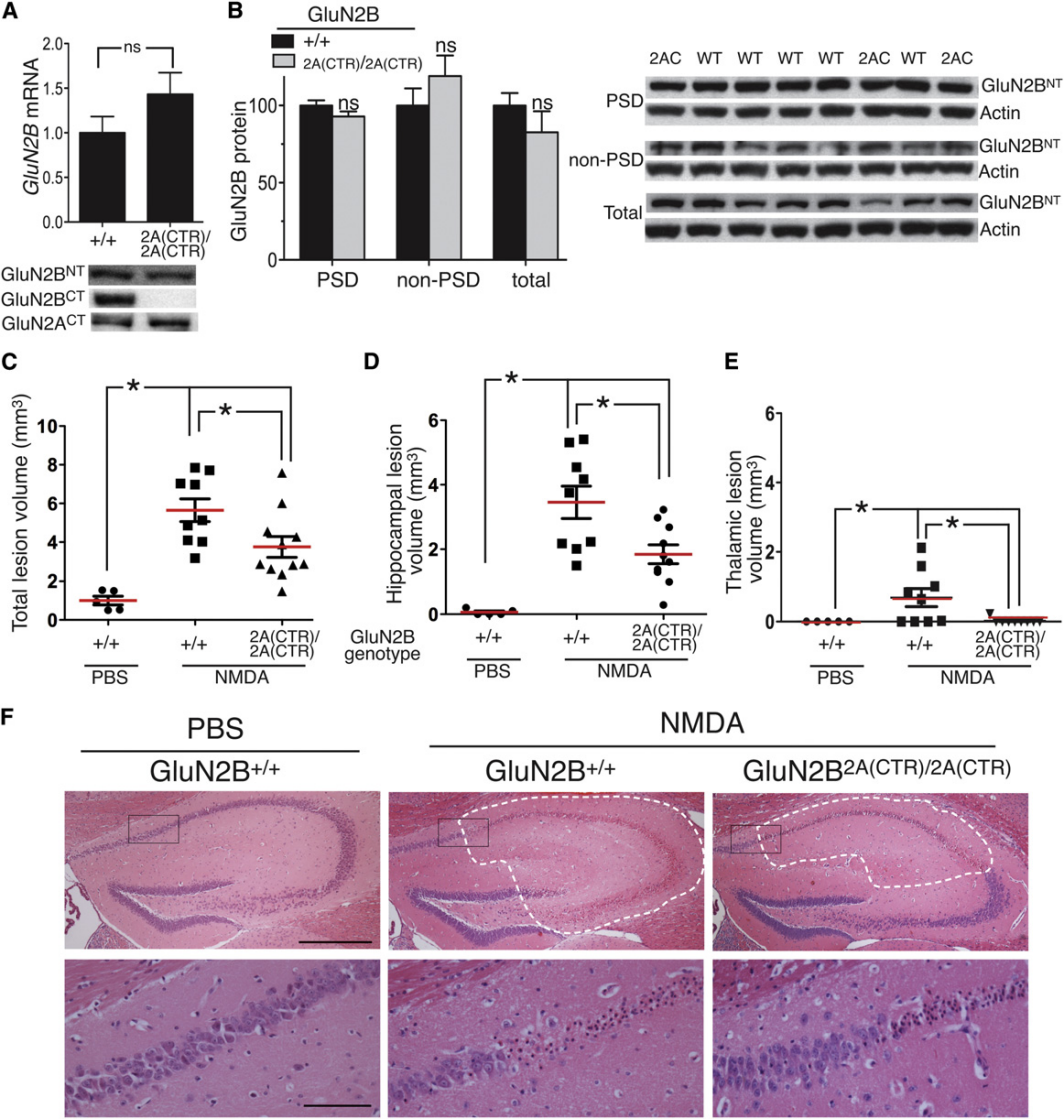
The GluN2 CTD Subtype Determines Excitotoxicity In Vivo (A) (Upper) GluN2B mRNA levels are not altered in forebrain of *GluN2B*^+/+^ versus *GluN2B*^2A(CTR)/2A(CTR)^ mice (n = 6). Mean ± SEM shown here and throughout the figure. (Lower) Example western illustrating equivalent GluN2B protein levels in homogenates of adult forebrains taken from *GluN2B*^+/+^ and *GluN2B*^2A(CTR)/2A(CTR)^ mice (t = −0.75; p = 0.51; n = 5). CT/NT = antibody to C/N-terminus of the indicated GluN2 subunit. (B) Levels of GluN2B protein are not altered in PSD-enriched or non-PSD-enriched fractions derived from synaptosomes prepared from homogenates of the adult hippocampus of *GluN2B*^+/+^ versus *GluN2B*^2A(CTR)/2A(CTR)^ mice. See Supplemental Experimental Procedures for details; n = 10 *GluN2B*^+/+^; n = 5 *GluN2B*^2A(CTR)/2A(CTR)^. (C–F) *GluN2B*^2A(CTR)/2A(CTR)^ mice exhibit smaller NMDA-induced lesion volumes. Brain lesion volumes (mm^3^) were calculated from hematoxylin-and-eosin-stained serial sections taken 24 hr following stereotaxic injection of 15 nmol NMDA into the hippocampus. (C–E) Total, hippocampal and thalamic lesion volumes were calculated (^∗^p < 0.05; ANOVA followed by Tukey's post hoc test; n = 9 (*GluN2B*^+/+^); n = 10; *GluN2B*^2A(CTR)/2A(CTR)^; n = 5; PBS-treated *GluN2B*^+/+^). (F) (Upper) Example pictures illustrating NMDA-induced damage in the hippocampus. White dashes indicate the boundary of the lesioned areas, identified by parenchymal pallor and vacuolation, and morphological neuronal changes (shrunken, triangulated nuclei and cytoplasm, eosinophilic neurons). Black boxes in the upper panel are shown in higher magnification in the lower panel to illustrate the lesion boundary in greater detail in the NMDA-injected mice. Upper and lower scale bars are 250 and 50 μm, respectively.

Despite the aforementioned similarities, we found one important difference; whole-cell NMDAR currents in *GluN2B*^2A(CTR)/2A(CTR)^ neurons were around 30% lower than *GluN2B*^+/+^ (Figure 2D). Levels of GluN2B protein were lower in DIV10 *GluN2B*^2A(CTR)/2A(CTR)^ cortical neurons (Figure S2H) and in P7 cortical protein extracts (Figure S2I; ruling out the possibility of an in vitro artifact). An explanation for this difference was found when we looked at GluN2B^2A(CTR)^ mRNA levels, which were lower both in DIV10 *GluN2B*^2A(CTR)/2A(CTR)^ cortical neurons and in P7 cortical extracts (Figures S2H and S2I). However, this decrement appeared to be a developmental-stage-dependent effect because by adulthood, levels of forebrain *GluN2B* mRNA (Figure 3A) and protein (p = 0.51, n = 5,5) were unaltered in *GluN2B*^+/+^ versus *GluN2B*^2A(CTR)/2A(CTR)^ mice. We hypothesize that GluN2B^2A(CTR)^, compared to wild-type GluN2B, may be transcribed, processed, or exported slightly less efficiently, which manifests itself in a mRNA decrement in development when expression of many genes, including those encoding NMDAR subunits, is changing rapidly.

To compare the effects of equivalent NMDAR activity in *GluN2B*^2A(CTR)/2A(CTR)^ and *GluN2B*^+/+^ neurons, we needed to adjust the concentration of applied NMDA to compensate for the lower currents in *GluN2B*^2A(CTR)/2A(CTR)^ neurons. A NMDA dose-response curve for both *GluN2B*^2A(CTR)/2A(CTR)^ and *GluN2B*^+/+^ neurons revealed no difference in their EC-50 s (Figure S2J). Based on these NMDA dose-responses, we predicted that an application of 17 and 21 μM NMDA to *GluN2B*^+/+^ neurons would induce the same current as an application of 30 and 50 μM, respectively, to *GluN2B*^2A(CTR)/2A(CTR)^ neurons (Figure 2E). This was then confirmed experimentally; application of 17 and 30 μM NMDA (hereafter NMDA_C1_) applied to *GluN2B*^+/+^ neurons and *GluN2B*^2A(CTR)/2A(CTR)^ neurons, respectively, induced equivalent currents (Figure 2F), as did application of the higher pair of NMDA concentrations: 21 and 50 μM NMDA (hereafter NMDA_C2_) applied to *GluN2B*^+/+^ neurons and *GluN2B*^2A(CTR)/2A(CTR)^, respectively (Figure 2F). Given that NMDAR-dependent excitotoxicity is predominantly Ca^2+^-dependent, we next studied the intracellular Ca^2+^ elevation triggered by NMDA_C1_ and NMDA_C2_. Treatment with NMDA_C1_ caused similar Ca^2+^ loads in *GluN2B*^2A(CTR)/2A(CTR)^ and *GluN2B*^+/+^ neurons, as did NMDA_C2_ (Figure 2G).

Satisfied that these doses of NMDA elicit equivalent NMDAR-dependent currents and Ca^2+^ loads, we next studied their effects on neuronal viability. Strikingly, we found that NMDA_C1_ and NMDA_C2_ both promoted more death in *GluN2B*^+/+^ neurons than in *GluN2B*^2A(CTR)/2A(CTR)^ (Figures 2H and 2I). Thus, swapping the *GluN2B* CTD for that of *GluN2A* in the mouse genome reduces the toxicity of NMDAR-dependent Ca^2+^ influx. This is in agreement with our studies based on the overexpression of GluN2A/2B-based wild-type and chimeric subunits (Figure 1), thus confirming the importance of the CTD subtype by two independent approaches. We also performed a similar set of experiments in DIV18 neurons. Because there remained a difference in whole-cell currents (around 25%), we again generated NMDAR current dose-response curves to allow us to pick pairs of NMDA concentrations (15 and 20 μM; 30 and 40 μM) which would trigger equivalent currents (Figure S2K). Consistent with our observations at DIV10, we once again saw increased NMDA-induced death in *GluN2B*^+/+^ neurons compared to *GluN2B*^2A(CTR)/2A(CTR)^ neurons experiencing equivalent levels of NMDAR activity (Figure S2L).

We next wanted to determine whether maximal levels of neuronal death could be achieved in neuronal populations devoid of CTD^2B^ if NMDAR activity were high enough. We treated *GluN2B*^2A(CTR)/2A(CTR)^ neurons with a high dose (100 μM) of NMDA and found that this triggered near-100% neuronal death, as it also did in *GluN2B*^+/+^ neurons (Figures 2H and 2I). Thus, the influence of excitotoxicity on the GluN2 CTD subtype is abolished when insults are very strong.

In the adult mouse forebrain, GluN2B and GluN2A are the major GluN2 NMDAR subunits (Rauner and Köhr, 2011; Sheng et al., 1994), raising the question as to whether the GluN2 CTD subtype (2A versus 2B) influences excitotoxicity in the adult forebrain in vivo. As stated above, adult forebrain GluN2B (protein and mRNA) levels are unaltered in *GluN2B*^+/+^ versus *GluN2B*^2A(CTR)/2A(CTR)^ mice (Figure 3A). We also specifically studied GluN2B levels in isolated protein fractions enriched in synaptic and peri/extrasynaptic NMDARs, following an established protocol (Milnerwood et al., 2010). Briefly, a synaptosomal preparation was made from the hippocampi of adult *GluN2B*^+/+^ and *GluN2B*^2A(CTR)/2A(CTR)^ mice. This prep was then split into a Triton-soluble “non-PSD enriched” fraction including extrasynaptic NMDARs, plus a Triton-insoluble (but SDS-soluble) “PSD-enriched” fraction containing synaptic NMDARs. We found no differences in the levels of GluN2B between *GluN2B*^+/+^ and *GluN2B*^2A(CTR)/2A(CTR)^ hippocampi with regard to either total homogenate, “Non-PSD enriched” fraction, or “PSD-enriched” fraction (Figure 3B). This biochemical data is in agreement with observations that the NMDAR:AMPAR current ratios in evoked EPSCs measured at holding potentials of −80 and +40 mV are not altered in adult CA1 pyramidal cells of GluN2B^2A(CTR)/2A(CTR)^ mutants compared to GluN2B^+/+^ controls (Thomas O'Dell, personal communication). Moreover, the decay time constant of NMDAR-mediated EPSCs recorded at +40 mV in GluN2B^2A(CRT)/2A(CTR)^ mutants was found to be indistinguishable from GluN2B^+/+^ controls (Thomas O'Dell, personal communication), indicative of a similar GluN2 subunit composition.

To promote excitotoxic neuronal loss, we stereotaxically injected a small (15 nmol) dose of NMDA into the hippocampus (just below the dorsal region of the CA1 layer) and quantified the resulting lesion volume 24 hr later. Consistent with the position of the injection site, the lesions were centered on the CA1 subregion, an effect potentially enhanced by the known vulnerability of this subregion to excitotoxic insults (Stanika et al., 2010). However the lesion also spread to other hippocampal subregions (CA3, dentate gyrus) as well as a small intrusion into the thalamus. Importantly, analysis revealed that *GluN2B*^2A(CTR)/2A(CTR)^ mice exhibited smaller lesion volumes in the hippocampus and the thalamic region (and smaller overall lesion volumes) than *GluN2B*^+/+^ mice (Figures 3C–3F). Thus, the GluN2 CTD subtype also influences NMDAR-mediated excitotoxicity in vivo.

### Differential Signaling to CREB Contributes to GluN2 CTD Subtype-Specific Excitotoxicity

**Figure 4:**
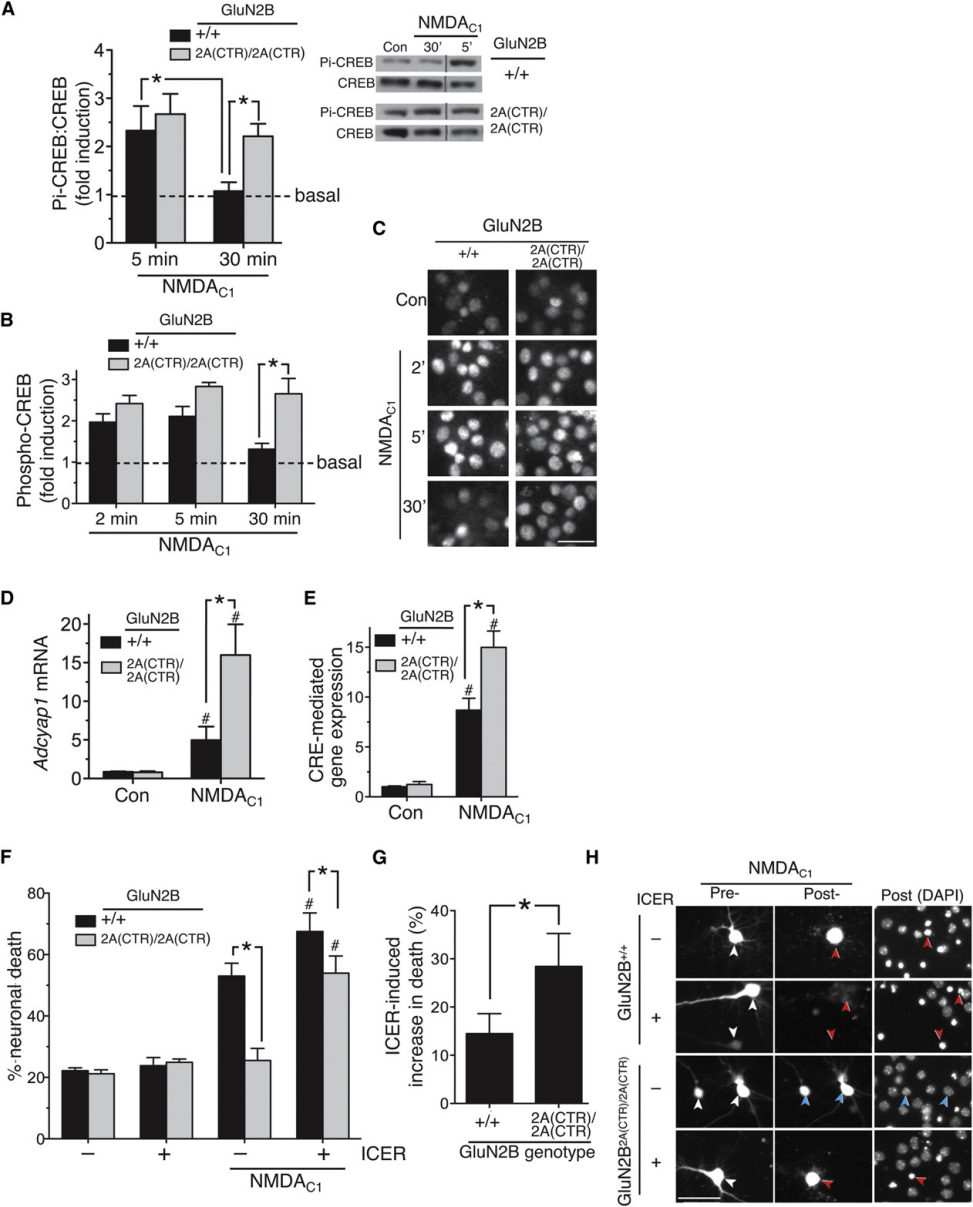
The GluN2 CTD Subtype Influences Excitotoxicity by Differential Coupling to a CREB Shut-Off Pathway (A) (Left) Quantitation of western blot analysis of phospho (serine-133)-CREB kinetics following NMDA_C1_ treatment, normalized to total CREB (^∗^p < 0.05; *GluN2B*^+/+^ n = 11; *GluN2B*^2A(CTR)/2A(CTR)^ n = 12). Mean ± SEM shown here and throughout the figure. (Right) Example blot (relevant samples within a single blot have been grouped). (B) Quantitation of immunohistochemical analysis of phospho-CREB kinetics following NMDA_C1_ treatment. (^∗^p < 0.05; n = 7 (*GluN2B*^+/+^); n = 4 (*GluN2B*^2A(CTR)/2A(CTR)^); 200 cells analyzed in each condition, in each repeat. (C) Example images relating to (B). At the 30 min time point, phospho-CREB levels remain high in *GluN2B*^2A(CTR)/2A(CTR)^ neurons but have returned to baseline in many *GluN2B*^+/+^ neurons. Scale bar = 30 μm. (D) NMDAR-mediated induction of the CREB target gene *Adcyap1* is elevated in *GluN2B*^2A(CTR)/2A(CTR)^ neurons, compared to *GluN2B*^+/+^ neurons. RNA was extracted at 4 hr posttreatment and subject to qPCR-based analysis of Adcyap1 (normalized to Gapdh; ^∗^p < 0.05; n = 5 (*GluN2B*^+/+^); n = 4 (*GluN2B*^2A(CTR)/2A(CTR)^). (E) NMDAR-mediated induction of CRE-dependent gene expression is elevated in *GluN2B*^2A(CTR)/2A(CTR)^ neurons, compared to *GluN2B*^+/+^ neurons. Neurons were transfected with a CRE-luciferase reporter plus pTK-renilla control and treated with NMDA_C1_ for 8 hr, after which CRE firefly reporter activity was assayed and normalized to renilla luciferase control (^∗^p < 0.05; n = 11 (*GluN2B*^+/+^); n = 12 (*GluN2B*^2A(CTR)/2A(CTR)^). (F) Effect of ICER expression on vulnerability to NMDAR-mediated excitotoxicity in *GluN2B*^+/+^ and *GluN2B*^2A(CTR)/2A(CTR)^ neurons. Neurons expressing eGFP plus either ICER1 or control vector (encoding β globin) were treated where indicated with NMDA_C1_. Images of cells were taken before and 24 hr post-NMDA treatment to track their fate, after which cells were fixed and nuclei DAPI stained. ^∗^p < 0.05 (indicated comparisons on figure); #p < 0.05 (comparing NMDA-treated ICER-expressing neurons with NMDA-treated globin-expressing neurons of that genotype), n = 9 (*GluN2B*^+/+^) and n = 11 (*GluN2B*^2A(CTR)/2A(CTR)^) NMDA_C1_-treated cultures were analyzed; 200–300 cells in total per condition/genotype combination. (G) ICER has a greater effect on vulnerability to excitotoxicity in *GluN2B*^2A(CTR)/2A(CTR)^ neurons compared to wild-type. From the data in (F), the difference between levels of NMDA-induced neuronal death ± ICER expression were calculated. ^∗^p < 0.05; n = 9 (*GluN2B*^+/+^); n = 11 (*GluN2B*^2A(CTR)/2A(CTR)^). (H) Example pictures from (F). White arrows indicate transfected neurons before NMDA treatment. Red/blue arrows in the “posttreatment” panels indicate dead/live cells, respectively. Scale bar 50 μm. See also Figure S3.

We next investigated the mechanistic basis for the observed GluN2 CTD subtype-dependent differences in vulnerability to excitotoxicity. NMDAR-dependent activation of CREB-dependent gene expression protects against excitotoxicity (Lee et al., 2005) and can act as a protective response to excitotoxic insults (Mabuchi et al., 2001). We found that basal levels of CREB (serine-133) phosphorylation (normalized to total CREB) were unaltered in *GluN2B*^2A(CTR)/2A(CTR)^ neurons (118% ± 12% compared to *GluN2B*^+/+^ neurons, p = 0.2). However we found that in response to treatment with NMDA_C1_, CREB (serine-133) phosphorylation was more prolonged in *GluN2B*^2A(CTR)/2A(CTR)^ neurons than in *GluN2B*^+/+^ neurons, as assayed by western blot and immunohistochemistry (Figures 4A–4C), and also that activation of a CRE-reporter gene and the CREB target gene *Adcyap1* was stronger in *GluN2B*^2A(CTR)/2A(CTR)^ neurons than *GluN2B*^+/+^ (Figures 4D and 4E). These differences did not extend to all transcriptional events: no differences were seen in the NMDA_C1_-induced activation of *Srxn1,* an AP-1 target gene (Soriano et al., 2009), or suppression of the FOXO target gene *Txnip* (Al-Mubarak et al., 2009; Figures S3A and S3B). To confirm whether CREB-dependent gene expression causally influenced vulnerability to NMDAR-mediated excitotoxicity we utilized the inhibitory CREB family member ICER which we have previously confirmed blocks the induction of CRE-mediated gene expression when expressed in cortical neurons (Papadia et al., 2005). ICER expression increased levels of NMDA_C1_-induced death in both *GluN2B*^2A(CTR)/2A(CTR)^ and *GluN2B*^+/+^ neurons (Figures 4F–4H). However, the effect of ICER on *GluN2B*^2A(CTR)/2A(CTR)^ neurons was greater than its effect on *GluN2B*^+/+^ neurons (Figure 4G), indicating that differential CREB activation is a contributing factor to the observed CTD subtype-dependent control of excitotoxicity.

**Figure 5:**
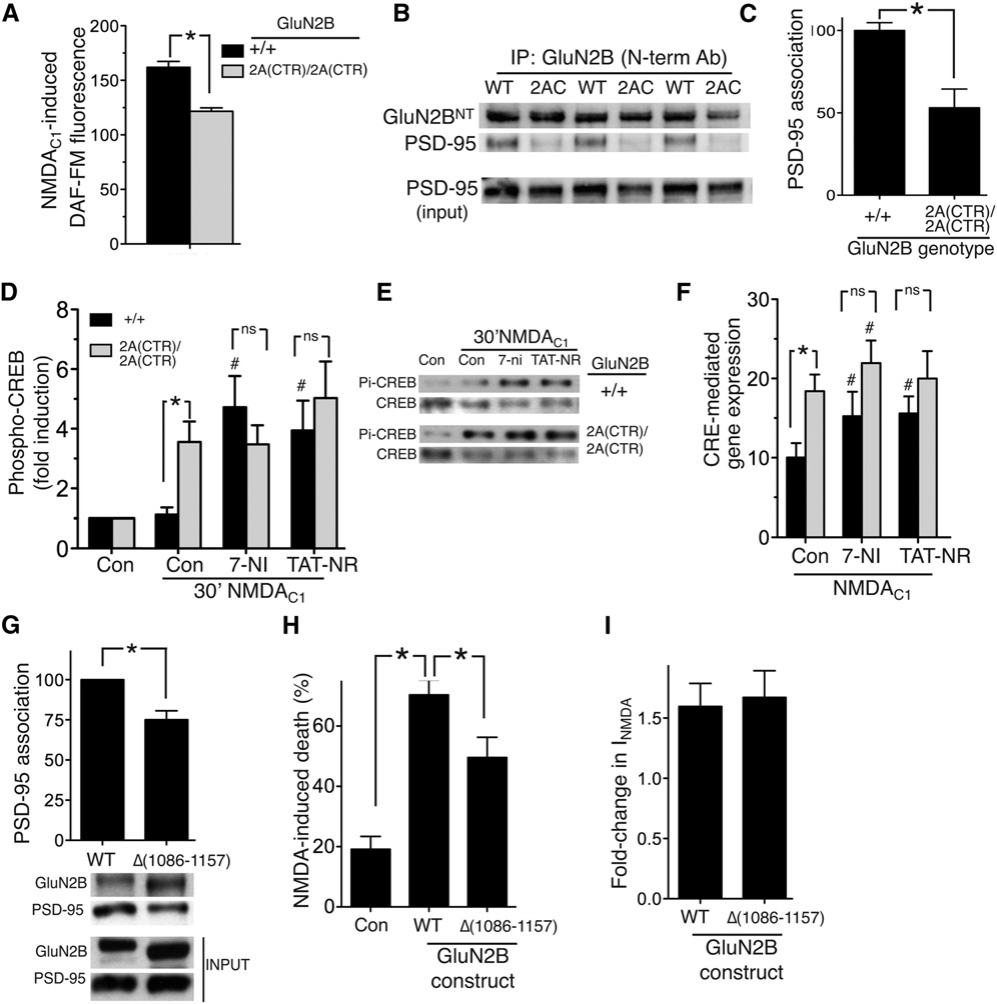
The GluN2B CTD Couples More Strongly to a PSD-95-nNOS-Mediated CREB Shut-Off Pathway Than that of GluN2A (A) DAF-FM-based NO assay (see Experimental Procedures) performed on neurons treated with NMDA_C1_ for 10 min. ^∗^p < 0.05; n = 6 (*GluN2B*^+/+^); n = 9 (*GluN2B*^2A(CTR)/2A(CTR)^). Mean ± SEM shown here and throughout the figure. (B and C) *GluN2B*^WT^ associates more strongly with PSD-95 than does *GluN2B*^2A(CTR)^. GluN2B was immunoprecipitated from *GluN2B*^+/+^(WT) and *GluN2B*^2A(CTR)/2A(CTR)^ (2AC) P7 cortical homogenates with a GluN2B N-terminal antibody. The presence of GluN2B and PSD-95 in the immunoprecipitate was analyzed by western blot, and the ratio of band intensities (PSD:GluN2B) was calculated (^∗^ p < 0.05; n = 11 (*GluN2B*^+/+^); n = 12 (*GluN2B*^2A(CTR)/2A(CTR)^). (D and E) Western analysis of CREB phosphorylation (normalized to total CREB) in neurons pretreated as indicated with 7-nitroindazole (5 μM) or TAT-NR2B9c (2 μM) prior to NMDA_C1_ treatment for 5 or 30 min. ^∗^, p < 0.05; n = 10 (*GluN2B*^+/+^); n = 8 (*GluN2B*^2A(CTR)/2A(CTR)^). #, p < 0.05 t test comparison of the effect of the drug, compared to the (NMDA-treated) control. (F) CRE reporter assay carried out as in Figure 4E. ^∗^p < 0.05; n = 5 (*GluN2B*^+/+^); n = 7 (*GluN2B*^2A(CTR)/2A(CTR)^). #, p < 0.05 paired t test comparison of the effect of the drug, compared to the control. (G) Deletion of the GluN2B CTD between 1086–1157 lowers GluN2B affinity for PSD-95. HEK cells were transfected with plasmids encoding GluN1, PSD-95, and GluN2B^WT^ or GluN2B^Δ(1086–1157)^. After 24 hr, protein was extracted, and the association of GluN2B or GluN2B^Δ(1086–1157)^ with PSD-95 was studied by coimmunoprecipitation, using an antibody to the N terminus of GluN2B. Upper, densitometric analysis of the resulting western blot (^∗^, p < 0.05 paired t test; n = 6). Lower, an example blot. (H) Deletion of the GluN2B CTD between 1086–1157 lowers GluN2B-mediated excitotoxicity. Neurons were transfected with the indicated GluN2B constructs or β-globin (plus eGFP marker), and NMDA-induced death was assessed as described in Figure 1D (^∗^p < 0.05 paired t test [n = 8]; 250–300 cells analyzed per condition). (I) Acute expression of GluN2B^WT^ or GluN2B^Δ(1086–1157)^ has a similar effect on NMDA-induced whole-cell currents. Neurons were transfected with the indicated constructs (plus eGFP marker), and whole-cell steady-state NMDAR-mediated currents evoked by 100 μM NMDA (normalized to cell capacitance) were compared to control-transfected neurons (β-globin; n = 4). See also Figure S4.

One known regulator of CREB phosphorylation is nitric oxide (NO) which is produced when NMDAR-dependent Ca^2+^ influx activates nNOS, recruited to the NMDAR signaling complex via PSD-95 association with GluN2 subunits (Aarts et al., 2002). Whereas basal NOS activity can contribute to CREB phosphorylation in dentate granule cells (Ciani et al., 2002), it has been found to suppress CREB phosphorylation in the hippocampus (Park et al., 2004; Zhu et al., 2006). Furthermore, nNOS inhibition or deficiency boosts CREB phosphorylation following stroke (Luo et al., 2007). Compared to *GluN2B*^2A(CTR)/2A(CTR)^ neurons, *GluN2B*^+/+^ neurons coupled more strongly to NMDA_C1_-induced NO production (Figure 5A), despite nNOS and PSD-95 levels being the same (Figures S4A and S4B). Moreover, nNOS inhibition by 7-nitroindazole treatment enhanced CREB phosphorylation and CREB-dependent gene expression more strongly in *GluN2B*^+/+^ neurons than *GluN2B*^2A(CTR)/2A(CTR)^ neurons, eliminating the CTD-subtype specific differences (Figures 5D–5F). This may be due to a stronger GluN2-PSD-95-nNOS coupling because association of GluN2B with PSD-95 was found to be stronger in P7 cortical extracts from *GluN2B*^+/+^ mice versus *GluN2B*^2A(CTR)/2A(CTR)^ mice (Figures 5B and 5C). Moreover, treatment of neurons with TAT-NR2B9c, which partly uncouples GluN2B from PSD-95 and NO production (Aarts et al., 2002), promoted more sustained CREB phosphorylation and enhanced CRE-reporter activity in NMDA_C1_-treated GluN2B^+/+^ neurons (Figures 5D–5F), but had little effect on these pathways in GluN2B^2A(CTR)/2A(CTR)^ neurons (with the caveat that TAT-NR2B9c disrupts GluN2B-PSD95 binding at lower concentrations than it does for GluN2A). Thus, CTD^2B^ couples mores strongly to PSD-95, NO production and nNOS-dependent CREB inactivation, enhancing vulnerability to excitotoxicity.

The basis for stronger association of PSD-95 with GluN2B^WT^ compared to GluN2B^2A(CTR)^ could be due to different sequences immediately upstream of the conserved C-terminal PDZ ligand. We generated a chimeric variant of GluN2B in which the final 12 amino acids of its CTD have been replaced by those of GluN2A (three amino acid differences, GluN2B^(2A-PDZ)^). Coimmunoprecipitation studies revealed that GluN2B^(2A-PDZ)^ had a similar affinity for PSD-95 as GluN2B (Figure S4C), indicating that immediate upstream sequence differences are not the basis for differential association of PSD-95 with the CTDs of GluN2B and GluN2A. Recently, additional PSD-95 interaction domains have been discovered on internal regions of CTD^2B^ (1086–1157; Cousins et al., 2009), which could contribute to the overall affinity of the CTD for PSD-95. The role of these additional regions in neurons is not yet clear, but could act to stabilize the primary interaction with the C-terminal PDZ ligand, or even act independently. Deletion of this region (creating GluN2B^Δ(1086–1157)^) resulted in a small reduction in PSD-95 association (Figure 5G). Importantly, NMDA-induced death following overexpression of GluN2B^Δ(1086–1157)^ in primary rat hippocampal neurons (as per the assays used in Figure 1) was significantly lower than in neurons overexpressing GluN2B^WT^ (Figure 5H), even though whole-cell NMDAR currents were found to be the same in GluN2B^Δ(1086–1157)^ as wild-type GluN2B^WT^-expressing neurons (Figure 5I), implicating this region of the CTD in contributing to prodeath NMDAR signaling.

## Discussion

We have demonstrated distinct roles for the CTDs of GluN2B and GluN2A in determining the dose response of NMDAR-mediated excitotoxicity. CTD^2B^ promotes neuronal death more efficiently than CTD^2A^, an effect which is observed regardless of whether the CTD is tethered to the channel portion of GluN2B or of GluN2A. Moreover, this difference is observed both in the context of acute chimeric subunit expression in wild-type neurons, as well as in a knockin mouse where the CTD is swapped at the genetic level. Using the latter approach, we demonstrated the influence of the GluN2 CTD subtype in controlling excitotoxic lesion volume in vivo. We also show that the GluN2 CTD subtype's ability to influence excitotoxicity is overcome when strong excitotoxic insults are applied.

These findings raise the question as to whether subunit composition (and CTD identity) underlies the known differential prodeath signaling from synaptic versus extrasynaptic NMDARs, or whether it represents an additional factor that influences excitotoxicity (Hardingham and Bading, 2010). Although some studies have reported that GluN2B is enriched at extrasynaptic sites (Groc et al., 2006; Martel et al., 2009; Tovar and Westbrook, 1999), apparently in favor of the first alternative, on closer inspection this study, plus published work, favors the latter alternative. Ca^2+^ influx dependent on intense *trans*-synaptic activation of synaptic NMDARs is well tolerated and neuroprotective (Hardingham and Bading, 2010; Hardingham et al., 2002; Léveillé et al., 2010; Zhang et al., 2011). In contrast, similar Ca^2+^ loads induced by the chronic activation of extrasynaptic NMDARs couple preferentially to prodeath pathways (Dick and Bading, 2010; Dieterich et al., 2008; Hardingham and Bading, 2010; Hardingham et al., 2002; Ivanov et al., 2006; Léveillé et al., 2008; Wahl et al., 2009; Xu et al., 2009; Zhang et al., 2007).

At developmental stages where GluN2B-containing NMDARs dominate at all locations, differential synaptic versus extrasynaptic NMDAR signaling is still observed (Hardingham et al., 2002). Importantly, the strong *trans*-synaptic activation of synaptic GluN2B-containg NMDARs is neuroprotective (Martel et al., 2009; Papadia et al., 2008). Our current study shows that the identity of the GluN2 CTD profoundly influences excitotoxicity in the context of chronic activation of all (synaptic and extrasynaptic) NMDARs, scenarios that are likely to exist in pathological situations such as ischemia, traumatic brain injury, or glutamate dyshomeostasis triggered by disease-causing agents. Thus, location/stimulus-specific effects can be uncoupled from GluN2 subunit-specific effects, suggesting that subunit/CTD composition represents an additional factor that determines the level of excitotoxicity following chronic NMDAR activation. This is further supported by the fact that recent electrophysiological and immuno-EM studies have shown that GluN2 subunit composition may not be dramatically different at synaptic versus extrasynaptic sites (Harris and Pettit, 2007; Petralia et al., 2010; Thomas et al., 2006). Our observations that swapping CTD^2B^ for CTD^2A^ has little effect on whether a subunit ends up at a synaptic or extrasynaptic site is consistent with the aforementioned studies reporting that subunits do not have a strong location preference. Any apparent enrichment of synaptic sites for GluN2A may reflect the fact that GluN2A upregulation coincides developmentally with increased synaptogenesis (Liu et al., 2004), or be due to the influence of sequences outside of the CTD.

That notwithstanding, GluN2B has been reported to be partly enriched at extrasynaptic locations in neurons (Groc et al., 2006; Martel et al., 2009; Tovar and Westbrook, 1999), which suggests that GluN2 subtype effects and location effects may cooperate to exacerbate excitotoxicity under certain circumstances. Of note, recent work has revealed a causal role for enhanced GluN2B-containing extrasynaptic NMDARs in ischemic neuronal death (Tu et al., 2010). Also, a specific increase in GluN2B-containing NMDARs in medium-sized spiny striatal neurons, specifically at extrasynaptic locations, contributes to phenotype onset in a model of Huntington's disease (Fan et al., 2007; Milnerwood et al., 2010), where the synaptic/extrasynaptic NMDAR balance controls mutant Huntingtin toxicity (Okamoto et al., 2009).

The idea that subunit composition influences excitotoxicity independently or additively to the influence of receptor location raises the possibility of a hierarchy of NMDARs when it comes to promoting excitotoxicity, based on the combination of composition (2A versus 2B) and location (synaptic versus extrasynaptic). Whereas strong activation of synaptic GluN2B-containing NMDARs is well-tolerated and neuroprotective (Martel et al., 2009; Papadia et al., 2008), the current study raises the possibility that activation of synaptic GluN2B-containing NMDARs (but not GluN2A-containing) could augment excitotoxicity in the context of chronic extrasynaptic NMDAR activation, for example, through enhanced NO production. This would explain the antiexcitotoxic effect of TAT-NR2B9c, PSD-95 knockdown, or disrupting the PSD-95-nNOS interface (Aarts et al., 2002; Cao et al., 2005; Sattler et al., 1999; Soriano et al., 2008b; Zhou et al., 2010), and the reversal of CTD^2B^-dependent CREB inactivation by TAT-NR2B9c and nNOS inhibition (Figure 5). However, because PSD-95 clusters have been observed at extrasynaptic sites (Carpenter-Hyland and Chandler, 2006), colocalizing with extrasynaptic NMDARs (Petralia et al., 2010), the possibility that extrasynaptic CTD^2B^ also contributes to this pathway should not be ruled out. Regardless of these issues, targeting GluN2B-PSD95 signaling to neurotoxic pathways offers genuine translational potential because it has been recently shown that stroke-induced damage and neurological deficits can be prevented in nonhuman primates by the administration of TAT-NR2Bc as late as 3 hr after stroke onset (Cook et al., 2012).

Investigations into why PSD-95 association with GluN2B^WT^ is stronger than its association with GluN2B^2A(CTR)^ implicated a previously identified internal region (Cousins et al., 2009) as a contributing factor, although deleting it had a relatively small effect on PSD-95 association, indicating that other determinants may also be relevant. Also, differing affinities of CTD^2B^ and CTD^2A^ for PSD-95 may be partly due to other factors binding CTD^2A^, occluding PSD-95 binding.

It is also possible that signals other than NO underlie the differential CTD subtype prodeath signaling, or that NO affects pathways other than CREB. One known NO target is the PI3K-Akt pathway, which is induced by NMDAR activity and neuroprotective in this context (Lafon-Cazal et al., 2002; Papadia et al., 2005). Modest NO levels promote PTEN S-nitrosylation, boosting Akt activity, whereas excessive NO also S-nitrosylates Akt itself, inactivating it (Numajiri et al., 2011). We have preliminary evidence that NMDA-induced Akt activation is enhanced in *GluN2B*^2A(CTR)/2A(CTR)^ neurons (M.A. Martel and G.E. Hardingham, unpublished data), and it will be of interest to determine any role of differential NO production. Also, it would be of interest to know whether NMDAR signaling to protective transcriptional responses other than CREB are sensitive to GluN2 CTD subtype (e.g., Iduna; Andrabi et al., 2011). These, and other issues surrounding subunit-specific signaling could benefit from a future systematic analysis of the NMDAR signaling complex in *GluN2B*^+/+^ versus *GluN2B*^2A(CTR)/2A(CTR)^ neurons.

## Experimental Procedures

### Neuronal Culture and Induction of Excitotoxicity

Cortical mouse and hippocampal rat neurons were cultured as described (Papadia et al., 2008) at a density of between 9 and 13 × 10^4^ neurons per cm^2^ from E17.5 mice or E21 rats with neurobasal growth medium supplemented with B27 (Invitrogen, Paisley, UK). Stimulations of cultured neurons were done in most cases after a culturing period of 9–11 days, during which neurons develop a network of processes, express functional NMDA-type and AMPA/kainate-type glutamate receptors, and form synaptic contacts. Other experiments were performed at DIV 18. To apply an excitotoxic insult, neurons were first placed overnight into a minimal-defined medium (Papadia et al., 2005) containing 10% MEM (Invitrogen) and 90% salt-glucose-glycine (SGG) medium (Bading et al., 1993; SGG: 114 mM NaCl, 0.219% NaHCO_3_, 5.292 mM KCl, 1 mM MgCl_2_, 2 mM CaCl_2_, 10 mM HEPES, 1 mM Glycine, 30 mM Glucose, 0.5 mM sodium pyruvate, 0.1% Phenol Red; osmolarity 325 mosm/l; Papadia et al., 2005). Neurons were then treated with NMDA (Tocris Bioscience, Bristol, UK) at the indicated concentrations for 1 hr, after which NMDARs were blocked by adding the antagonist MK-801 (10 μM). After a further 23 hr, neurons were fixed and subjected to DAPI staining, and cell death was quantified by counting (blind) the number of shrunken, pyknotic nuclei as a percentage of the total. For analysis of excitotoxicity in *GluN2B*^+/+^ versus *GluN2B*^2A(CTR)/2A(CTR)^ neurons, approximately 800–1,200 cells were analyzed per condition, per replicate (repeated across several replicates).

### GluN2B-2A(CTR) Knockin Mouse

GluN2B-2A(CTR) knockin mice contain a GluN2B gene in which the protein coding portion of the C-terminal exon has been replaced with the protein coding region of the C-terminal exon of GluN2A (C-terminal domain replacement, CTR). The C-terminal exon encodes amino acids 867G to 1482V (GluN2B) and 866G to 1464V (GluN2A), which represents over 95% of the CTD, beginning at position 838E (GluN2A) and 839E (GluN2B). All other regions of the GluN2B gene are unaltered, including the 3′UTR, although there remains a 61 bp insert containing a loxP site located after the STOP codon at the beginning of the 3′UTR (a remnant of the excision of the Neo-selection cassette). To obtain cultured neurons from GluN2B^2A(CTR)/2A(CTR)^ mice, male and female heterozygous GluN2B^+/2A(CTR)^ mice were mated, and the cortices from individual E17.5 mice were cultured as above. See Supplemental Experimental Procedures for further details.

### Transfection and Following the Fate of Transfected Cells

Neurons were transfected at DIV8 using Lipofectamine 2000 (Invitrogen), using an established protocol (McKenzie et al., 2005). Transfection efficiency was approximately 5%. Greater than 99% of eGFP-expressing transfected neurons were NeuN-positive, and <1% were GFAP-positive (Soriano et al., 2008a), confirming their neuronal identity. For studying the effects of expressing wild-type and chimeric receptors based on GluN2A and GluN2B, constructs were cotransfected with peGFP (ratio 1:1) to identify transfected cells. Coexpression at this ratio was confirmed in the case of pRFP (Papadia et al., 2008). After 48 hr, the transfected neurons were then either subjected to electrophysiological analysis or their fate following an excitotoxic insult was studied. Pictures of GFP-expressing neurons were taken on a Leica AF6000 LX imaging system, with a DFC350 FX digital camera. Using the automated cell-finder function within the Leica AF6000 software, images of transfected neurons were taken both before and 24 hr after a 1 hr treatment with NMDA (20 μM). Cell death was assessed by counting the number of surviving GFP-positive neurons. In the vast majority of cases, death was easily spotted as an absence of a healthy GFP-expressing cell where one once was. In place of the cell, there was in most cases (>90%) evidence of death in the form of fragmented neurites, fluorescent cell debris, and a pyknotic nucleus (Papadia et al., 2008). This confirmed that the cells were genuinely dying as opposed to more unlikely scenarios, such as quenching of eGFP fluorescence in a subpopulation of neurons. For each condition, 150–200 neurons were studied over several independent experiments. An identical experimental regime was employed for studying the influence of ICER expression on vulnerability of GluN2B^2A(CTR)/2A(CTR)^ and GluN2B^+/+^ neurons to NMDA-induced excitotoxicity. Neurons were transfected with vectors encoding eGFP and the inhibitory CREB family member ICER1 (Stehle et al., 1993), or a control vector (encoding β-globin). We have previously confirmed that ICER1 expression inhibits CRE-mediated gene expression in neurons (Papadia et al., 2005). The fate of transfected neurons following exposure to NMDA was then studied as described previously.

### Analysis of Extrasynaptic NMDAR Currents

To measure extrasynaptic NMDAR currents, synaptically located NMDARs were blocked by quantal activation-mediated blockade by MK-801, as previously described (Martel et al., 2009; Papadia et al., 2008). Briefly, whole-cell NMDAR currents were recorded (100 μM NMDA, in Mg^2+^-free and TTX/PTX-containing recording solution), after which the agonist was washed-out the recording chamber for 2 min. Irreversible NMDAR open-channel blocker MK-801 (10 μM; Tocris Bioscience) was then applied for 10 min, effectively antagonizing NMDARs located at the synapse and experiencing the localized, quantal presynaptic glutamate release (Martel et al., 2009; Nakayama et al., 2005). Following the 10 min incubation period, MK-801 was then washed out (2 min), and the resulting extrasynaptic NMDAR currents were acquired.

### Other Procedures

See Supplemental Experimental Procedures for details of genotyping, plasmid generation, electrophysiological recording conditions, qPCR analysis, Ca^2+^ imaging, stereotaxic NMDA administration, NO assays, western blotting and immunofluorescence, co-immunoprecipitation, and equipment settings. All procedures were authorized under a UK Home Office approved project licence and adhered to regulations specified in the Animals (Scientific Procedures) Act (1986) and approved by the University of Edinburgh's Local Ethical Review Committee. Statistical testing involved a 2-tailed paired Student's t test. For studies employing multiple testing, we used a one-way ANOVA followed by Fisher's LSD or Tukey's post hoc test.

## Supplemental Information

Document S1. Figures S1–S4 and Supplemental Experimental Procedures

## References

[bib1] Aarts M., Liu Y., Liu L., Besshoh S., Arundine M., Gurd J.W., Wang Y.T., Salter M.W., Tymianski M. (2002). Treatment of ischemic brain damage by perturbing NMDA receptor- PSD-95 protein interactions. Science.

[bib2] Al-Mubarak B., Soriano F.X., Hardingham G.E. (2009). Synaptic NMDAR activity suppresses FOXO1 expression via a cis-acting FOXO binding site: FOXO1 is a FOXO target gene. Channels.

[bib3] Andrabi S.A., Kang H.C., Haince J.F., Lee Y.I., Zhang J., Chi Z., West A.B., Koehler R.C., Poirier G.G., Dawson T.M., Dawson V.L. (2011). Iduna protects the brain from glutamate excitotoxicity and stroke by interfering with poly(ADP-ribose) polymer-induced cell death. Nat. Med..

[bib4] Bading H., Ginty D.D., Greenberg M.E. (1993). Regulation of gene expression in hippocampal neurons by distinct calcium signaling pathways. Science.

[bib5] Berberich S., Punnakkal P., Jensen V., Pawlak V., Seeburg P.H., Hvalby O., Köhr G. (2005). Lack of NMDA receptor subtype selectivity for hippocampal long-term potentiation. J. Neurosci..

[bib6] Berliocchi L., Bano D., Nicotera P. (2005). Ca^2+^ signals and death programmes in neurons. Philos. Trans. R. Soc. Lond. B Biol. Sci..

[bib7] Cao J., Viholainen J.I., Dart C., Warwick H.K., Leyland M.L., Courtney M.J. (2005). The PSD95-nNOS interface: a target for inhibition of excitotoxic p38 stress-activated protein kinase activation and cell death. J. Cell Biol..

[bib8] Carpenter-Hyland E.P., Chandler L.J. (2006). Homeostatic plasticity during alcohol exposure promotes enlargement of dendritic spines. Eur. J. Neurosci..

[bib9] Choi D.W. (1988). Glutamate neurotoxicity and diseases of the nervous system. Neuron.

[bib10] Choi D.W. (1992). Excitotoxic cell death. J. Neurobiol..

[bib11] Ciani E., Guidi S., Bartesaghi R., Contestabile A. (2002). Nitric oxide regulates cGMP-dependent cAMP-responsive element binding protein phosphorylation and Bcl-2 expression in cerebellar neurons: implication for a survival role of nitric oxide. J. Neurochem..

[bib12] Cook D.J., Teves L., Tymianski M. (2012). Treatment of stroke with a PSD-95 inhibitor in the gyrencephalic primate brain. Nature.

[bib13] Cousins S.L., Kenny A.V., Stephenson F.A. (2009). Delineation of additional PSD-95 binding domains within NMDA receptor NR2 subunits reveals differences between NR2A/PSD-95 and NR2B/PSD-95 association. Neuroscience.

[bib14] Cull-Candy S., Brickley S., Farrant M. (2001). NMDA receptor subunits: diversity, development and disease. Curr. Opin. Neurobiol..

[bib15] Dick O., Bading H. (2010). Synaptic activity and nuclear calcium signaling protect hippocampal neurons from death signal-associated nuclear translocation of FoxO3a induced by extrasynaptic N-methyl-D-aspartate receptors. J. Biol. Chem..

[bib16] Dieterich D.C., Karpova A., Mikhaylova M., Zdobnova I., König I., Landwehr M., Kreutz M., Smalla K.H., Richter K., Landgraf P. (2008). Caldendrin-Jacob: a protein liaison that couples NMDA receptor signalling to the nucleus. PLoS Biol..

[bib17] Fan M.M., Raymond L.A. (2007). N-methyl-D-aspartate (NMDA) receptor function and excitotoxicity in Huntington's disease. Prog. Neurobiol..

[bib18] Fan M.M., Fernandes H.B., Zhang L.Y., Hayden M.R., Raymond L.A. (2007). Altered NMDA receptor trafficking in a yeast artificial chromosome transgenic mouse model of Huntington's disease. J. Neurosci..

[bib19] Frizelle P.A., Chen P.E., Wyllie D.J.A. (2006). Equilibrium constants for (R)-[(S)-1-(4-bromo-phenyl)-ethylamino]-(2,3-dioxo-1,2,3,4-tetrahydroquinoxalin-5-yl)-methyl]-phosphonic acid (NVP-AAM077) acting at recombinant NR1/NR2A and NR1/NR2B N-methyl-D-aspartate receptors: Implications for studies of synaptic transmission. Mol. Pharmacol..

[bib20] Furukawa H., Singh S.K., Mancusso R., Gouaux E. (2005). Subunit arrangement and function in NMDA receptors. Nature.

[bib21] Graham D., Darles G., Langer S.Z. (1992). The neuroprotective properties of ifenprodil, a novel NMDA receptor antagonist, in neuronal cell culture toxicity studies. Eur. J. Pharmacol..

[bib22] Groc L., Heine M., Cousins S.L., Stephenson F.A., Lounis B., Cognet L., Choquet D. (2006). NMDA receptor surface mobility depends on NR2A-2B subunits. Proc. Natl. Acad. Sci. USA.

[bib23] Hardingham G.E., Fukunaga Y., Bading H. (2002). Extrasynaptic NMDARs oppose synaptic NMDARs by triggering CREB shut-off and cell death pathways. Nat. Neurosci..

[bib24] Hardingham G.E., Bading H. (2010). Synaptic versus extrasynaptic NMDA receptor signalling: implications for neurodegenerative disorders. Nat. Rev. Neurosci..

[bib25] Harris A.Z., Pettit D.L. (2007). Extrasynaptic and synaptic NMDA receptors form stable and uniform pools in rat hippocampal slices. J. Physiol..

[bib26] Hatton C.J., Paoletti P. (2005). Modulation of triheteromeric NMDA receptors by N-terminal domain ligands. Neuron.

[bib27] Ivanov A., Pellegrino C., Rama S., Dumalska I., Salyha Y., Ben-Ari Y., Medina I. (2006). Opposing role of synaptic and extrasynaptic NMDA receptors in regulation of the extracellular signal-regulated kinases (ERK) activity in cultured rat hippocampal neurons. J. Physiol..

[bib28] Lafon-Cazal M., Perez V., Bockaert J., Marin P. (2002). Akt mediates the anti-apoptotic effect of NMDA but not that induced by potassium depolarization in cultured cerebellar granule cells. Eur. J. Neurosci..

[bib29] Lau A., Tymianski M. (2010). Glutamate receptors, neurotoxicity and neurodegeneration. Pflugers Arch..

[bib30] Lee B., Butcher G.Q., Hoyt K.R., Impey S., Obrietan K. (2005). Activity-Dependent Neuroprotection and CREB: Kinase Coupling, Stimulus Intensity, and Temporal Regulation of CREB Phosphorylation at Serine 133. J. Neurosci..

[bib31] Léveillé F., El Gaamouch F., Gouix E., Lecocq M., Lobner D., Nicole O., Buisson A. (2008). Neuronal viability is controlled by a functional relation between synaptic and extrasynaptic NMDA receptors. FASEB J..

[bib32] Léveillé F., Papadia S., Fricker M., Bell K.F., Soriano F.X., Martel M.A., Puddifoot C., Habel M., Wyllie D.J., Ikonomidou C. (2010). Suppression of the intrinsic apoptosis pathway by synaptic activity. J. Neurosci..

[bib33] Lipton S.A. (2006). Paradigm shift in neuroprotection by NMDA receptor blockade: memantine and beyond. Nat. Rev. Drug Discov..

[bib34] Liu X.B., Murray K.D., Jones E.G. (2004). Switching of NMDA receptor 2A and 2B subunits at thalamic and cortical synapses during early postnatal development. J. Neurosci..

[bib35] Liu Y., Wong T.P., Aarts M., Rooyakkers A., Liu L., Lai T.W., Wu D.C., Lu J., Tymianski M., Craig A.M., Wang Y.T. (2007). NMDA receptor subunits have differential roles in mediating excitotoxic neuronal death both in vitro and in vivo. J. Neurosci..

[bib36] Luo C.X., Zhu X.J., Zhou Q.G., Wang B., Wang W., Cai H.H., Sun Y.J., Hu M., Jiang J., Hua Y. (2007). Reduced neuronal nitric oxide synthase is involved in ischemia-induced hippocampal neurogenesis by up-regulating inducible nitric oxide synthase expression. J. Neurochem..

[bib37] Mabuchi T., Kitagawa K., Kuwabara K., Takasawa K., Ohtsuki T., Xia Z., Storm D., Yanagihara T., Hori M., Matsumoto M. (2001). Phosphorylation of cAMP response element-binding protein in hippocampal neurons as a protective response after exposure to glutamate in vitro and ischemia in vivo. J. Neurosci..

[bib38] Martel M., Wyllie D.J., Hardingham G.E. (2009). In developing hippocampal neurons, NR2B-containing N-methyl-D-aspartate receptors (NMDARs) can mediate signaling to neuronal survival and synaptic potentiation, as well as neuronal death. Neuroscience.

[bib39] McKenzie G.J., Stevenson P., Ward G., Papadia S., Bading H., Chawla S., Privalsky M., Hardingham G.E. (2005). Nuclear Ca2+ and CaM kinase IV specify hormonal- and Notch-responsiveness. J. Neurochem..

[bib40] Milnerwood A.J., Gladding C.M., Pouladi M.A., Kaufman A.M., Hines R.M., Boyd J.D., Ko R.W., Vasuta O.C., Graham R.K., Hayden M.R. (2010). Early increase in extrasynaptic NMDA receptor signaling and expression contributes to phenotype onset in Huntington's disease mice. Neuron.

[bib41] Monyer H., Burnashev N., Laurie D.J., Sakmann B., Seeburg P.H. (1994). Developmental and regional expression in the rat brain and functional properties of four NMDA receptors. Neuron.

[bib42] Nakayama K., Kiyosue K., Taguchi T. (2005). Diminished neuronal activity increases neuron-neuron connectivity underlying silent synapse formation and the rapid conversion of silent to functional synapses. J. Neurosci..

[bib43] Neyton J., Paoletti P. (2006). Relating NMDA receptor function to receptor subunit composition: limitations of the pharmacological approach. J. Neurosci..

[bib44] Numajiri N., Takasawa K., Nishiya T., Tanaka H., Ohno K., Hayakawa W., Asada M., Matsuda H., Azumi K., Kamata H. (2011). On-off system for PI3-kinase-Akt signaling through S-nitrosylation of phosphatase with sequence homology to tensin (PTEN). Proc. Natl. Acad. Sci. USA.

[bib45] Okamoto S., Pouladi M.A., Talantova M., Yao D., Xia P., Ehrnhoefer D.E., Zaidi R., Clemente A., Kaul M., Graham R.K. (2009). Balance between synaptic versus extrasynaptic NMDA receptor activity influences inclusions and neurotoxicity of mutant huntingtin. Nat. Med..

[bib46] Olney J.W. (1969). Brain lesions, obesity, and other disturbances in mice treated with monosodium glutamate. Science.

[bib47] Paoletti P. (2011). Molecular basis of NMDA receptor functional diversity. Eur. J. Neurosci..

[bib48] Papadia S., Soriano F.X., Léveillé F., Martel M.A., Dakin K.A., Hansen H.H., Kaindl A., Sifringer M., Fowler J., Stefovska V. (2008). Synaptic NMDA receptor activity boosts intrinsic antioxidant defenses. Nat. Neurosci..

[bib49] Papadia S., Stevenson P., Hardingham N.R., Bading H., Hardingham G.E. (2005). Nuclear Ca2+ and the cAMP response element-binding protein family mediate a late phase of activity-dependent neuroprotection. J. Neurosci..

[bib50] Park C., Shin K.S., Ryu J.H., Kang K., Kim J., Ahn H., Huh Y. (2004). The inhibition of nitric oxide synthase enhances PSA-NCAM expression and CREB phosphorylation in the rat hippocampus. Neuroreport.

[bib51] Petralia R.S., Wang Y.X., Hua F., Yi Z., Zhou A., Ge L., Stephenson F.A., Wenthold R.J. (2010). Organization of NMDA receptors at extrasynaptic locations. Neuroscience.

[bib52] Rauner C., Köhr G. (2011). Triheteromeric NR1/NR2A/NR2B receptors constitute the major N-methyl-D-aspartate receptor population in adult hippocampal synapses. J. Biol. Chem..

[bib53] Ryan T.J., Emes R.D., Grant S.G., Komiyama N.H. (2008). Evolution of NMDA receptor cytoplasmic interaction domains: implications for organisation of synaptic signalling complexes. BMC Neurosci..

[bib54] Sattler R., Xiong Z., Lu W.Y., Hafner M., MacDonald J.F., Tymianski M. (1999). Specific coupling of NMDA receptor activation to nitric oxide neurotoxicity by PSD-95 protein. Science.

[bib55] Sheng M., Cummings J., Roldan L.A., Jan Y.N., Jan L.Y. (1994). Changing subunit composition of heteromeric NMDA receptors during development of rat cortex. Nature.

[bib56] Soriano F.X., Léveillé F., Papadia S., Higgins L.G., Varley J., Baxter P., Hayes J.D., Hardingham G.E. (2008). Induction of sulfiredoxin expression and reduction of peroxiredoxin hyperoxidation by the neuroprotective Nrf2 activator 3H-1,2-dithiole-3-thione. J. Neurochem..

[bib57] Soriano F.X., Martel M.A., Papadia S., Vaslin A., Baxter P., Rickman C., Forder J., Tymianski M., Duncan R., Aarts M. (2008). Specific targeting of pro-death NMDA receptor signals with differing reliance on the NR2B PDZ ligand. J. Neurosci..

[bib58] Soriano F.X., Baxter P., Murray L.M., Sporn M.B., Gillingwater T.H., Hardingham G.E. (2009). Transcriptional regulation of the AP-1 and Nrf2 target gene sulfiredoxin. Mol. Cells.

[bib59] Stanika R.I., Winters C.A., Pivovarova N.B., Andrews S.B. (2010). Differential NMDA receptor-dependent calcium loading and mitochondrial dysfunction in CA1 vs. CA3 hippocampal neurons. Neurobiol. Dis..

[bib60] Stehle J.H., Foulkes N.S., Molina C.A., Simonneaux V., Pévet P., Sassone-Corsi P. (1993). Adrenergic signals direct rhythmic expression of transcriptional repressor CREM in the pineal gland. Nature.

[bib61] Thomas C.G., Miller A.J., Westbrook G.L. (2006). Synaptic and extrasynaptic NMDA receptor NR2 subunits in cultured hippocampal neurons. J. Neurophysiol..

[bib62] Tovar K.R., Westbrook G.L. (1999). The incorporation of NMDA receptors with a distinct subunit composition at nascent hippocampal synapses *in vitro*. J. Neurosci..

[bib63] Traynelis S.F., Wollmuth L.P., McBain C.J., Menniti F.S., Vance K.M., Ogden K.K., Hansen K.B., Yuan H., Myers S.J., Dingledine R. (2010). Glutamate receptor ion channels: structure, regulation, and function. Pharmacol. Rev..

[bib64] Tu W., Xu X., Peng L., Zhong X., Zhang W., Soundarapandian M.M., Balel C., Wang M., Jia N., Zhang W. (2010). DAPK1 interaction with NMDA receptor NR2B subunits mediates brain damage in stroke. Cell.

[bib65] von Engelhardt J., Coserea I., Pawlak V., Fuchs E.C., Köhr G., Seeburg P.H., Monyer H. (2007). Excitotoxicity in vitro by NR2A- and NR2B-containing NMDA receptors. Neuropharmacology.

[bib66] Wahl A.S., Buchthal B., Rode F., Bomholt S.F., Freitag H.E., Hardingham G.E., Rønn L.C., Bading H. (2009). Hypoxic/ischemic conditions induce expression of the putative pro-death gene Clca1 via activation of extrasynaptic N-methyl-D-aspartate receptors. Neuroscience.

[bib67] Weitlauf C., Honse Y., Auberson Y.P., Mishina M., Lovinger D.M., Winder D.G. (2005). Activation of NR2A-containing NMDA receptors is not obligatory for NMDA receptor-dependent long-term potentiation. J. Neurosci..

[bib68] Xu J., Kurup P., Zhang Y., Goebel-Goody S.M., Wu P.H., Hawasli A.H., Baum M.L., Bibb J.A., Lombroso P.J. (2009). Extrasynaptic NMDA receptors couple preferentially to excitotoxicity via calpain-mediated cleavage of STEP. J. Neurosci..

[bib69] Zhang S.J., Steijaert M.N., Lau D., Schütz G., Delucinge-Vivier C., Descombes P., Bading H. (2007). Decoding NMDA receptor signaling: identification of genomic programs specifying neuronal survival and death. Neuron.

[bib70] Zhang S.J., Buchthal B., Lau D., Hayer S., Dick O., Schwaninger M., Veltkamp R., Zou M., Weiss U., Bading H. (2011). A signaling cascade of nuclear calcium-CREB-ATF3 activated by synaptic NMDA receptors defines a gene repression module that protects against extrasynaptic NMDA receptor-induced neuronal cell death and ischemic brain damage. J. Neurosci..

[bib71] Zhou L., Li F., Xu H.B., Luo C.X., Wu H.Y., Zhu M.M., Lu W., Ji X., Zhou Q.G., Zhu D.Y. (2010). Treatment of cerebral ischemia by disrupting ischemia-induced interaction of nNOS with PSD-95. Nat. Med..

[bib72] Zhu X.J., Hua Y., Jiang J., Zhou Q.G., Luo C.X., Han X., Lu Y.M., Zhu D.Y. (2006). Neuronal nitric oxide synthase-derived nitric oxide inhibits neurogenesis in the adult dentate gyrus by down-regulating cyclic AMP response element binding protein phosphorylation. Neuroscience.

