# Supplementary material for: The Subtype of GluN2 C-terminal Domain Determines the Response to Excitotoxic Insults
Source: Neuron. 2012 May 10;74(3):543–56. doi: 10.1016/j.neuron.2012.03.021 (PMC3398391; doi:10.1016/j.neuron.2012.03.021)
Supplement: Document S1. Figures S1–S4 and Supplemental Experimental Procedures [file mmc1.pdf]

Neuron, volume *74*

Supplemental Information

The Subtype of GluN2 C-terminal Domain Determines the Response to Excitotoxic Insults

Marc-Andre Martel, Tomás J. Ryan, Karen F. S. Bell, Jill H. Fowler, Aoife McMahon, Bashayer Al-Mubarak, Noboru H. Komiyama, Karen Horsburgh, Peter C. Kind, Seth G.N. Grant, David J.A. Wyllie, and Giles E. Hardingham

Inventory of Supplemental Information

1. Supplemental Figures
-Figure S1 relates to Figure 1 in the main text.
-Figure S2 relates to Figure 2 in the main text.
-Figure S3 relates to Figure 4 in the main text.
-Figure S4 relates to Figure 5 in the main text.

2. Supplemental Experimental Procedures

3. Supplemental References

1.Supplemental Data: Figures S1-S4

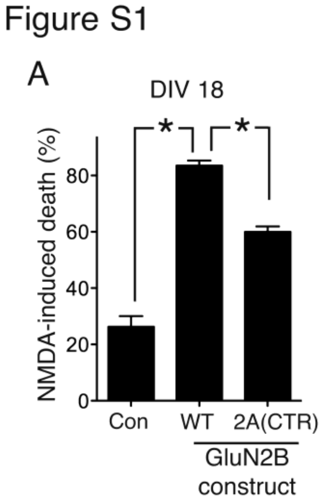

Fig. S1, related to Fig. 1. A) In DIV18 neurons GluN2B^{WT} expression renders neurons more vulnerable to an excitotoxic insult (15 µM NMDA for 1h), but replacing the CTD to that of GluN2A reduces the level of toxicity (*P<0.05, n=4, 120-160 cells analysed per condition)

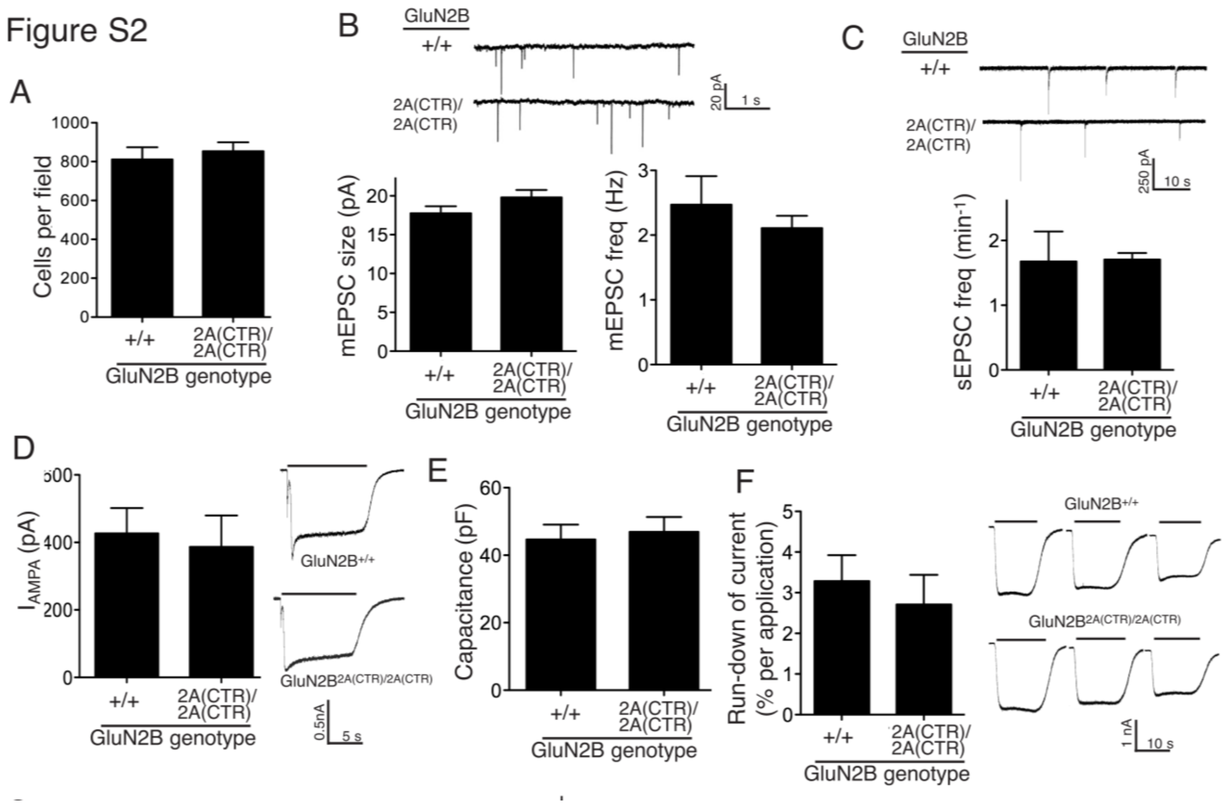

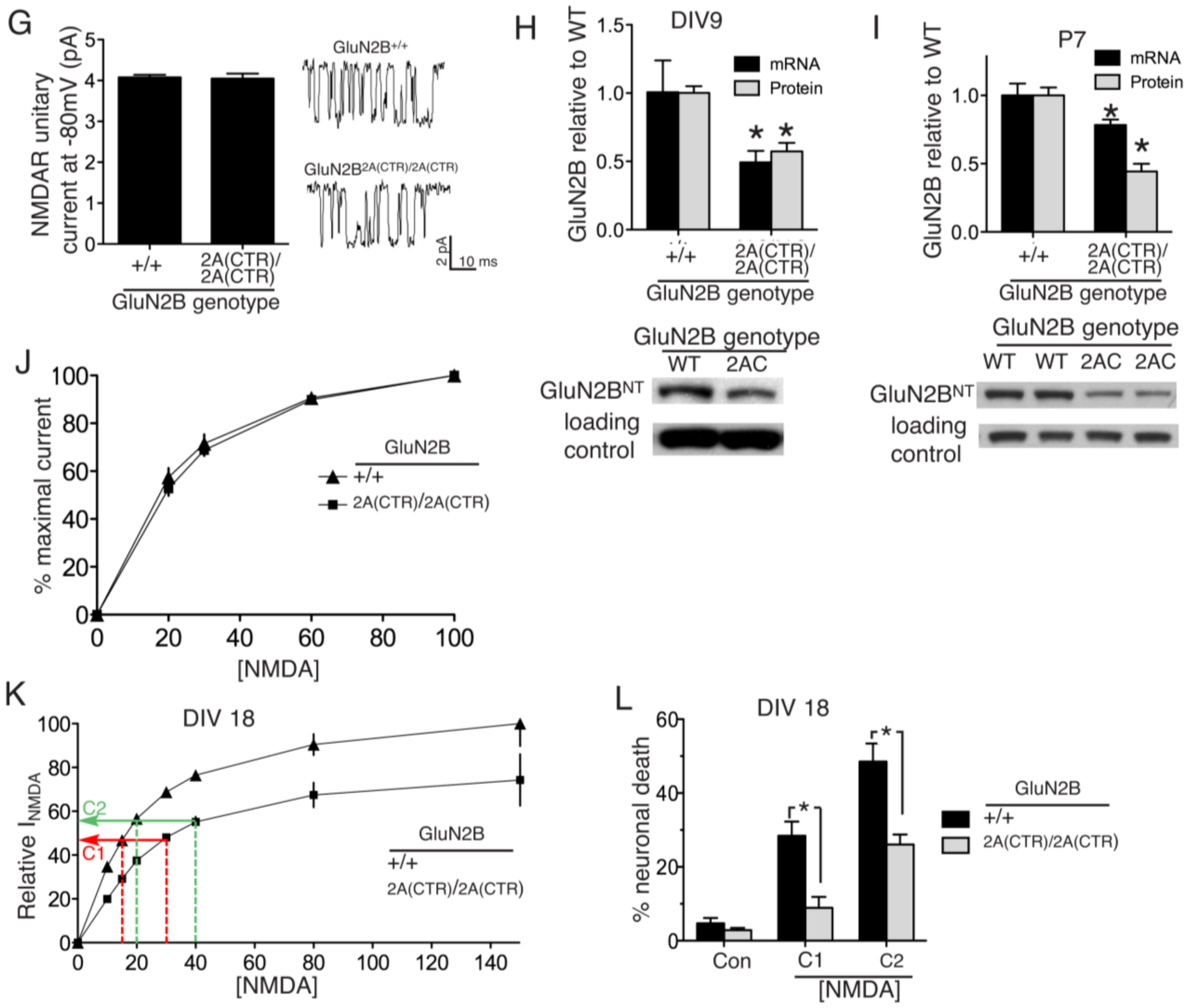

Fig. S2, related to Fig. 2. A) *GluN2B*$^{+/+}$ and *GluN2B*$^{2A(CTR)/2A(CTR)}$ cortical neurons show similar levels of basal viability. Cultures were plated at the same density (9-13 x 10^4 neurons per cm^2) and the density of viable neurons analysed at DIV9 (n=8 (*GluN2B*$^{+/+}$), n=12 (*GluN2B*$^{2A(CTR)/2A(CTR)}$). **B)** *GluN2B*$^{+/+}$ and *GluN2B*$^{2A(CTR)/2A(CTR)}$ cortical neurons exhibit mEPSCs of similar size and frequency. Graphs show quantitation (25 cells over n=4 cultures (*GluN2B*$^{+/+}$), 27 cells over n=4 cultures(*GluN2B*$^{2A(CTR)/2A(CTR)}$). Example is shown (upper)**. C)** *GluN2B*$^{+/+}$ and *GluN2B*$^{2A(CTR)/2A(CTR)}$ cortical neurons show similar levels of spontaneous synaptic activity (sEPSC frequency). Graphs show quantitation (17 cells over n=7 cultures (*GluN2B*$^{+/+}$), 17 cells over n=6 cultures (*GluN2B*$^{2A(CTR)/2A(CTR)}$). Example trace is shown (upper)**. D)** *GluN2B*$^{+/+}$ and *GluN2B*$^{2A(CTR)/2A(CTR)}$ cortical neurons show similar AMPA receptor currents. Left graph shows quantitation (21 cells over n=7 cultures (*GluN2B*$^{+/+}$), 24 cells over n=6 cultures (*GluN2B*$^{2A(CTR)/2A(CTR)}$). S-AMPA applied at 50 µM. Example traces shown (right). **E)** *GluN2B*$^{+/+}$ and *GluN2B*$^{2A(CTR)/2A(CTR)}$ cortical neurons show similar capacitances; 21 cells over n=7 cultures (*GluN2B*$^{+/+}$), 22 cells over n=6 cultures (*GluN2B*$^{2A(CTR)/2A(CTR)}$). **F)** Baseline whole-cell NMDAR currents evoked by 100 µM NMDA were measured, then 10 further 10 second applications of NMDA were carried out, spread over 10 minutes. The average % run-down of currents per application was then calculated for each cell (n=7 per genotype). Example traces show 2nd, 5th and 10th applications. **G)** Single-channel analysis of NMDA-evoked events in *GluN2B*$^{+/+}$ and *GluN2B*$^{2A(CTR)/2A(CTR)}$ cortical neurons. Approximately 250 events were analysed per genotype within n=3 independent cultures. Mean unitary currents were calculated for each culture and the mean taken from the 3 independent cultures. **H,I)** GluN2B protein levels measured (F, n=10 (both genotypes)) and P7 cortex (G, n=9 (both genotypes), normalized to loading control (ß globin). *p<0.05 (two-tailed t-Test). **J)** Same data as shown in Fig. 2E, but currents normalized to the maximum current for that genotype (n=8 cells per genotype) to illustrates that the EC-50s for NMDA are not significantly different. **K)** Calculation of NMDA

concentrations (C1 and C2) predicted to trigger equivalent NMDAR currents in *GluN2B*$^{+/+}$ and *GluN2B*$^{2A(CTR)/2A(CTR)}$ DIV18 neurons, based on dose response curves (n=13 *GluN2B*$^{+/+}$ cells, n=12 *GluN2B*$^{2A(CTR)/2A(CTR)}$ cells). Relative NMDAR currents are expressed as a % of the maximum current obtained in *GluN2B*$^{+/+}$ neurons. **L)** NMDA-induced cell death is diminished in DIV18 neurons containing *GluN2B*$^{2A(CTR)}$ compared to *GluN2B*^{WT}. Neurons were treated for 1 h with $NMDA_{C1}$ or $NMDA_{C2}$. Cell death was analysed after 24 h (*p<0.05, n=6 (*GluN2B*$^{+/+}$), n=7 (*GluN2B*$^{2A(CTR)/2A(CTR)}$, 5,000-10,000 cells analysed per treatment per genotype).

Figure S3

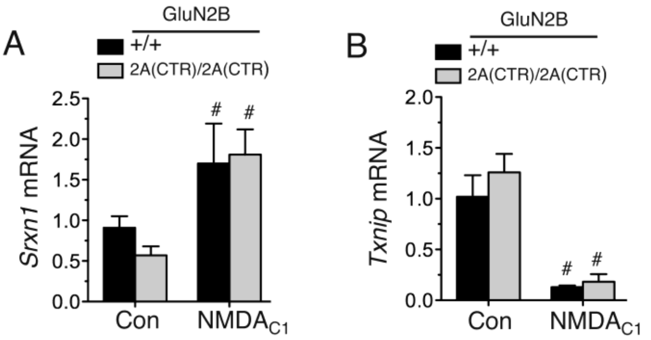

Fig. S3, related to Fig. 4. A,B) NMDAR-dependent induction of the AP-1 target gene Srxn1 is similar in *GluN2B*$^{+/+}$ and *GluN2B*$^{2A(CTR)/2A(CTR)}$ cortical neurons, as is NMDAR-dependent suppression of the FOXO target gene Txnip. RNA was extracted at 4 h post-treatment and subject to qPCR-based analysis of Srxn1 and Txnip levels (normalised to Gapdh, #p<0.05 compared to unstimulated, n=5 (*GluN2B*$^{+/+}$), n=4 (*GluN2B*$^{2A(CTR)/2A(CTR)}$).

Figure S4

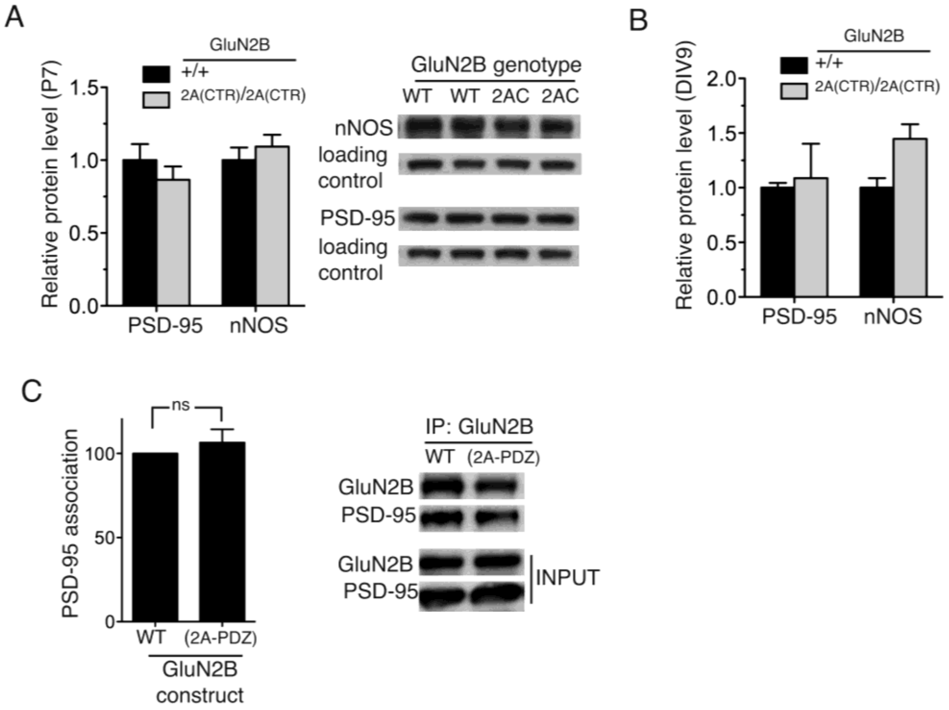

Fig. S4, related to Fig. 5. A,B) *GluN2B*$^{+/+}$ and *GluN2B*$^{2A(CTR)/2A(CTR)}$ cortical neurons show similar levels of PSD-95 and nNOS expression in P7 cortices (C, n=9 (*GluN2B*$^{+/+}$), n=9 (*GluN2B*$^{2A(CTR)/2A(CTR)}$) and DIV10 cultures (D, n=12 (*GluN2B*$^{+/+}$), n=7 (*GluN2B*$^{2A(CTR)/2A(CTR)}$)). **C) HEK cells were transfected with plasmids encoding GluN1, PSD-95 and** GluN2B^{WT} or GluN2B$^{(2A-PDZ)}$. After 24 h, protein was extracted and association of GluN2B/GluN2B$^{(2A-PDZ)}$ with PSD-95 studied by co-immunoprecipitation, using a GluN2B N-terminal antibody. Left: densitometric analysis of the resulting western blot (n=3 (GluN2B^{WT}), n=7 (GluN2B($^{2A-PDZ)}$)). Right: example blot.

2. Supplemental Experimental Procedures

Plasmids

Plasmids encoding for GluN2A with its cytoplasmic C-terminal domain replaced with that of GluN2B (GluN2A$^{2B(CTR)}$) and of GluN2B with its C-terminal domain replaced with that of GluN2A (GluN2B$^{2A(CTR)}$) were based on pCis-GluN2A and pCis-GluN2B (Rutter and Stephenson, 2000) and created as follows. Although the CTD of GluN2A and GluN2B begin at 838E (GluN2A) and 839E (GluN2B), the cloning strategy (see below) involved swapping a piece beginning in the M4 region (at position 825/826). To enable the CTDs to be exchanged, a NotI site was introduced into position 2474 (GluN2A) and the equivalent position of GluN2B (position 2477). This alteration left the amino acid sequence of GluN2A unchanged but introduced a single amino acid change in GluN2B (glycine to alanine) at position 826 at the end of the final trans-membrane domain which did not affect the expression or function of the subunits. Nevertheless, all chimeric receptors were compared to "wild-type" receptors that contained this mutation. For GluN2A$^{2B(CTR)}$, fragment GluN2A(1-824) was obtained by digestion of pCis-GluN2A with EcoRI and NotI (Promega, Southampton, UK); concurrently, pCis-GluN2B was also digested with EcoRI and NotI to obtain the complementary fragment pCis-GluN2B(826-1482). The DNA fragments were purified from the agarose gel using QIAquick Gel Extraction kit (Qiagen UK). Relevant fragments were ligated overnight at 4 °C by mixing 6 µl of GluN2A(1-824), 2 µl of pCis-GluN2B(826-1482), 1 µl of T4 DNA ligase (Agilent Technologies UK Ltd) and 1 µl of T4 DNA ligase Buffer. To construct GluN2B$^{2A(CTR)}$: fragment GluN2B(1-825) was isolated with ClaI (Promega) and NotI; fragment GluN2A(825-1464) by restriction enzymes NotI and EcoRI; and the vector pCis was obtained with ClaI and EcoRI. The purified DNAs were then ligated using 3.5 µg GluN2B(1-825), 3.5 µg GluN2A(825-1464), 1 µl pCis, 1 µl T4 DNA ligase and 1 µl of T4 Buffer. The resulting ligation reactions were then transformed in JM109 competent cells, and subsequent steps followed the same procedure as for the mutagenesis reaction. To generate pCis-GluN2B$^{(2A\text{-}PDZ)}$, pCis-GluN2B^{WT} was mutated by employing the technique of PCR-based amplification of pCis-GluN2B^{WT} using 5'-phosphorylated primers with the desired mismatches in one of the primers: 5' GGA CAT GTT TAT AAG AAA ATG CCT AGT ATT GAG TC 3', 5' ATT GCT GGA GCC ATT GAA AGC 3'. The resulting PCR product was ligated 2h at room temperature (NEB ligase) and transformed into JM-109 cells for expression of the plasmid. The final construct was sequenced to verify the incorporation of the amino acid mutations (VY**E**K**LS**SIESDV to VY**K**K**MP**SIESDV). The GluN2B$^{\Delta(1086\text{-}1157)}$ construct was created by generating two fragments of the original plasmid using two different primer pairs: 5' ATG CGG CCG CAG TGA TGA CTT CAA GCG AGA TTC 3' with 5' GCT AGG CAC CGG TTG TAA C 3', and 5' ATG CGG CCG CCC TTA TAT TGC TGC TTC CTC CTC TTG 3' with 5' GGG ATG ATC AGT GCT TGC TTC 3'. Each fragment comprised a common ClaI site and inserted a NotI site after K1086 and before R1158, respectively for each reaction. The amplification product of the two reactions was digested with ClaI + NotI and ran on an agarose gel. The relevant DNA fragments were then isolated and ligated (using a 1:1 ratio of each fragment) as described above to yield GluN2B$^{\Delta(1086\text{-}1157)}$, in which the amino acids 1087 to 1157 were effectively replaced by two glycines. All constructs were verified by sequencing (SBS sequencing service, University of Edinburgh) to confirm the correct identity of the final plasmid sequences. Other plasmids used in the study are CRE-firefly luciferase, pTK-Renilla both Promega), pSV-ICER1 (Stehle et al., 1993).

Electrophysiological recording and analysis

Coverslips containing hippocampal or cortical neurons DIV 8-11 were transferred to a recording chamber perfused (at a flow rate of 3-5 ml/min) with an external recording solution composed of (in mM): 150 NaCl, 2.8 KCl, 10 HEPES, 2 $CaCl_2$, 1 $MgCl_2$, 10 glucose, 0.1 glycine and 0.002 strychnine, pH 7.3 (320-330 mOsm). Patch-pipettes were made from thick-walled borosilicate glass (Harvard Apparatus, Kent, UK) and filled with a K-gluconate-based internal solution containing (in mM): 155 K-gluconate, 2 MgCl2, 10 Na-HEPES, 10 Na-PiCreatine, 2 Mg_2-ATP and 0.3 Na_3-GTP,

pH 7.3 (300 mOsm). Electrode tips were fire-polished for a final resistance ranging between 5-10 MΩ. Currents were recorded at room temperature (21 ± 2°C) using either an Axopatch-1C or Axon Multiclamp 700B amplifier (Molecular Devices, Union City, CA). Neurons were voltage-clamped at –60 mV, and recordings were rejected if the holding current was greater than –100 pA or if the series resistance drifted by more than 20% of its initial value (<30 MΩ). Whole-cell currents were analyzed using WinEDR v3.2 software (John Dempster, University of Strathclyde, UK).

To assess spontaneous EPSC (sEPSC) frequency, 5 to 6 min of continuous recordings were obtained. The frequency was then calculated as the number of EPSCs greater than 150 pA (to exclude mEPSCs) and separated in time by more than 1 sec, over the duration of the recording. For miniature EPSC (mEPSC) frequency analysis, 5-10 min recordings of at least 300 events were made in presence of 0.3 µM tetrodotoxin and 50 µM picrotoxin (TTX/PTX, from Tocris Bioscience, Bristol, UK). To measure AMPA receptor-mediated currents, 50 µM S-AMPA (Tocris Bioscience) was applied for 5-10 sec to reach a steady-state plateau, washed out for 1-2 min, then re-applied to elicit a second response. The same procedure was used to acquire whole-cell NMDAR currents (100 µM NMDA, Tocris Bioscience), except using Mg^{2+}-free external recording solution in which MgCl2 was substituted with 2 mM NaCl. When evaluating ifenprodil sensitivity of the NMDAR currents, ifenprodil (3 µM, Tocris Bioscience) was added for 2 min and 2 additional NMDA applications were performed to measure the remaining NMDAR current. All currents were quantified as a 1 sec average of the steady-state plateau minus the baseline at the second agonist application, and normalised to the cell capacitance.

Single-channel currents from $GluN2B^{+/+}$ or $GluN2B^{2A(CTR)}$ neurons were recorded in outside-out membrane patches excised from the cell soma. Single-channel activity, at –80 mV, was elicited by NMDA (10 µM) in the presence of glycine (100 µM). Analysis of the amplitudes of events was carried out using WinEDR 3.2 software and open point amplitude histograms of events with durations greater than 3 ms were fitted with a single Gaussian component to determine the mean amplitude of events in each recording. The chord conductances reported assume a reversal potential of 0 mV.

GluN2B-2A(CTR) mouse generation, breeding and genotyping

GluN2B-2A(CTR) knock-in mice contain a GluN2B gene in which the protein coding portion of the C-terminal exon has been replaced with the protein coding portion of the C-terminal exon of GluN2A (C-Terminal domain Replacement=CTR). This region encodes amino acids 867G to 1482V (GluN2B) and 866G to 1464V (GluN2A), which represents over 95% of the CTD (which begins at position 838E (GluN2A) and 839E (GluN2B). Briefly, the GluN2B-2A(CTR) targeting vector was created through the alteration of a previously constructed *GluN2B* targeting vector constructed from a mouse DNA library (acquired from R. Sprengel and P. Seeburg, Heidelberg). The 5' end of the homology arm of the GluN2B-2A(CTR) targeting vector is made up of 5.89kb of the GluN2B gene (100% identity), beginning with part of intron 12-13, then the whole of exon 13, intron13-14, exon 14 and intron14-15. 3' of this homology arm is the sequence encoding the protein coding portion of the GluN2A C-terminal exon, followed by a loxP-flanked PGKEM7-Neomycin selection cassette, followed by 1.78kb of the GluN2B 3'UTR (i.e. the non-coding region of GluN2B exon 15) which comprises the 3' homology arm. The GluN2B-2A(CTR) targeting vector was introduced into E14Tg2A mouse embryonic stem cells to target the GluN2B locus in. Successfully targeted clones were injected into blastocysts and implanted into pseudopregnant mice. Male chimeras were crossed onto C57BL/6J females to obtain F1 progeny, and germline transmission of the GluN2B-2A(CTR) locus confirmed in those animals subsequently crossed onto transgenic mice expressing Cre recombinase under a CMV promoter in order to excise the Neo selection cassette. Following excision, the only sequence replaced at the GluN2B locus in the working GluN2B-2A(CTR) mouse colony is the protein-coding portion of the C-terminal exon of GluN2B. This has been replaced with the protein-coding portion of the C-terminal exon of GluN2A. The large (2.3 kb) 3'UTR sequence of GluN2B is unaltered at the GluN2B-2A(CTR) locus except for the existence of a 61 bp insert containing a loxP site located after the STOP codon at the beginning of the 3'UTR (a remnant of the excision of the Neo-selection cassette). The altered locus was verified by

sequencing, and generation of the predicted protein product in mice confirmed by western blotting with antibodies specific for the N-terminal of GluN2B, the CTD of GluN2B, and the CTD of GluN2A. To obtain cultured neurons from GluN2B$^{2A(CTR)/2A(CTR)}$ mice, male and female heterozygous GluN2B$^{+/2A(CTR)}$ mice were mated and the cortices from individual E17.5 mice cultured as above. Normal Mendelian ratios of genotypes were observed and GluN2B$^{2A(CTR)/2A(CTR)}$ neurons were compared in all experiments to GluN2B$^{+/+}$ neurons cultured from wild-type littermates. Genotyping reactions were performed with the following primers: A = 5'-TCA GTG CTT GCT TCA CGG CAG C-3', B = 5'-CTC CTC TCC AGC CTC CCA CAC T-3', C = 5'-CCA CAC GTA CGG GGA TGA CCA-3'. Primer pair A-B recognized the wild-type allele, amplifying a product of 570 bp, whereas primer pair C-B gave a product of approximately 649 bp from the mutant allele (see Fig. 2a(i)).

In vivo stereotaxic injection of NMDA

All procedures were authorised under a UK Home Office approved project licence and adhered to regulations specified in the Animals (Scientific Procedures) Act (1986). GluN2A wild type and chimera mice (2-5 month old 75-165 days old, 24-30g) were anaesthetised with an intraperitoneal injection of avertin (tribromoethanol, Sigma, 250mg/kg) and transferred to a stereotaxic frame (Kopf). Rectal temperature was monitored throughout the surgical procedure and maintained at 37°C by the use of heating lamps. A midline incision was made, the skull exposed and a small burrhole made using a dental drill at the appropriate coordinates over the hippocampus (AP -2mm and ML −1.5 mm from Bregma using the atlas of Paxinos and Franklin, 2001). A blunt-ended needle attached to a 26 gauge 2 µl Hamilton syringe was slowly lowered into the hippocampus to a depth of 1.5 mm from the dural surface. Mice received intrahippocampal injections of 0.5µl of NMDA (15nmol; GluN2B$^{+/+}$ n=9; GluN2B$^{2A(CTR)/2A(CTR)}$ n=10) or vehicle (PBS, GluN2B$^{+/+}$ n=5) at the rate of 0.1µl per minute. The needle was left in place for a further 10 min to allow for diffusion of the injectate, then the incision was sutured and mice were returned to their cages for a further 24 hours. Mice were deeply anaesthetised with 5% isoflurane then perfused transcardially with 0.9% heparinised saline followed by 4% paraformaldehyde in 50 mM phosphate buffer. Whole brains were post-fixed in 4% paraformaldehyde before undergoing automated tissue processing through a series of alcohols and were paraffin embedded.

Serial coronal sections (10 µm) were cut and 2 sections were retained every 120 µm for haematoxylin and eosin (H&E) staining. Neuronal damage was defined by the presence of shrinkage and triangulation of the nucleus and cytoplasm, increased eosinophilia of the cytoplasm surrounded by vacuolation and pallor in the neuropil layer. Areas of neuronal damage were transcribed onto copies of atlas sections from every third coronal level throughout the extent of the lesion (Paxinos and Franklin, 2001). Areas of neuronal damage (mm^2) were calculated using a calibrated image analysis system (MCID M4, Imaging Research, St-Catherine, Canada). The volume of tissue damage in individual animals (mm^3) was calculated by plotting the areas of neuronal damage in each section against the corresponding anterior–posterior atlas co-ordinate and calculating the area under the curve.

RNA isolation, RT-PCR and qPCR.

RNA was isolated using the Qiagen RNeasy isolation reagents (including the optional DNase treatment) following passage of the cells through a QiaShredder column. For qPCR, cDNA was synthesized from 1-3 µg RNA using the Stratascript QPCR cDNA Synthesis kit (Stratagene, Amsterdam, Netherlands) according to the manufacturer's instructions. Briefly, the required amount of RNA (up to 3 µg) was diluted in RNase-free water (up to 7 µl final volume) and mixed on ice with 2x cDNA Synthesis master mix (10 µl), random primers: oligo-dT primers 3:1 (total 2 µl- 200 ng) and either 1 µl RT/RNase block enzyme mixture (for RT reactions) or 1 µl water (for No RT control reactions). Reaction mixtures were mixed and span down and incubated for 2 min at 25°C, 40 min at 42 °C and 5 min at 95°C. cDNA was stored at -20°C.

Dilutions of this cDNA were subsequently used for real-time PCR (cDNA equivalent to 6 ng of initial RNA per 15 µl qPCR reaction for all genes. qPCR was performed in an Mx3000P

QPCR System (Stratagene) using Brilliant SYBR Green QPCR Master Mix (Stratagene) according to the manufacturer's instructions. Briefly, the required amount of template was mixed on ice with 2x Brilliant SYBR Green Master Mix, forward and reverse primers at 200 nM each final concentration, 30 nM final concentration ROX passive reference dye and water to the required reaction volume. Technical replicates as well as no template and no RT negative controls were included and at least 3 biological replicates were studied in each case. The sequences of the primers used are as follows: *Gapdh* forward, 5'-GGG TGT GAA CCA CGA GAA AT-3'; *Gapdh* reverse, 5'-CCT TCC ACA ATG CCA AAG TT-3'; *Txnip* forward, 5'-GGA AAC AAA TAT GAG TAC AAG TTC G-3'; *Txnip* reverse, 5'-CCA TTG GCA AGG TAA GTG TG-3'; *Adcyap* forward, 5'-ATG TGT AGC GGA GCA AGG-3'; Adcyap reverse, 5'-GAG TGG CGT TTG GTA AGG-3'; *Srxn1* forward, 5'-GAC GTC CTC TGG ATC AAA G-3'; *Srxn1* reverse, 5'-GCA GGA ATG GTC TCT CTC TG-3'; *GluN2B* forward, 5'-GTG AGG TGG TCA TGA AGA GG-3'; *GluN2B* reverse, 5'-GCT AGG CAC CGG TTG TAA C-3'. The cycling programme was 10 min. at 95 °C; 40 cycles of 30 sec at 95 °C, 40 sec at 60°C with detection of fluorescence and 30 sec at 72 °C; 1 cycle (for dissociation curve) of 1 min at 95 °C and 30 sec at 55 °C with a ramp up to 30 sec at 95 °C (ramp rate: 0.2°C/sec) with continuous detection of fluorescence on the 55-95 °C ramp. The data were analysed using the MxPro QPCR analysis software (Stratagene). Expression of the gene of interest was normalized to Gapdh.

Calcium imaging

Ca^{2+} imaging was performed as described (Hardingham et al., 1997; Soriano et al., 2008) at 37ºC in aCSF (150 mM NaCl, 3 mM KCl, 10 mM HEPES, 2 mM CaCl2, 1mM $MgCl_2$, 1mM glucose). Briefly, cells were loaded with 11µM Fluo-3 AM (from a stock solution of 2.2 mM Fluo-3 dissolved in anhydrous DMSO containing 20% (w/v) Pluronic detergent) for 30 min at 37°C. Fluo-3 fluorescence images (excitation 472 ± 15 nm, emission 520 ± 15 nm) were taken at one frame per 5 sec using a Leica AF6000 LX imaging system, with a DFC350 FX digital camera. To calibrate images, Fluo-3 was saturated by adding 50 µM ionomycin to the perfusion chamber (to obtain F_{max}) and quenched with 10 mM $MnCl_2$ + 50 µM ionomycin to levels corresponding to 100 nM Ca^{2+} (Minta et al., 1989), which was in turn used to calculate F_{min}. Free Ca^{2+} concentrations were calculated from fluorescence signal (F) according to the equation $[Ca^{2+}] = Kd(F - F_{min})/(F_{max} - F)$, and expressed as a multiple of the Kd of Fluo-3 (which is approximately 315 nM). In order to quantitate the effect of $NMDA_{C1}$ and $NMDA_{C2}$ on intracellular $[Ca^{2+}]$ in $GluN2B^{2A(CTR)/2A(CTR)}$ and $GluN2B^{+/+}$ neurons, average $[Ca^{2+}]$ was calculated 5 sec before and 60-95 sec after application of NMDA. For each genotype/treatment 90-105 cells were analysed within 3 independent experiments.

Reporter assays

Firefly luciferase-based reporter gene constructs (CRE-Luc) were transfected along with a renilla expression vector (pTK-RL). Neurons were stimulated (where appropriate) 24 h after transfection (for 8 h). Luciferase assays were performed using the Dual Glo assay kit (Promega) with Firefly luciferase-based reporter gene activity normalized to the Renilla control (pTK-RL plasmid) in all cases.

NO assay

Neurons were loaded with 5 µM DAF-FM DA (4-amino-5-methylamino-2',7'-difluorofluorescein diacetate) for 30 min at 37ºC followed by washing four times and incubation at 37ºC to enable the DAF-FM DA to hydrolyse in the cell. Neurons were then treated with NMDA for the indicated times and lysed in ice-cold PBS+ 0.5% NP-40. DAF-FM fluorescence in a portion of the lysate was measured in a fluorometer: excitation 475-490 nm, emission 510-550 nm (FLUOstar OPTIMA, BMG Labtech, Aylesbury, UK) and normalized to cell number (Cell Titer Glo Assay, Promega) measured from a portion of the lysate.

PSD fractionation, western blotting and immunofluorescence

To isolate PSD-enriched proteins (containing synaptic NMDARs) and "Non-PSD enriched" (containing extrasynaptic NMDARs) we used an approach previously described for studying synaptic and extrasynaptic NMDAR populations (Milnerwood et al., 2010). Briefly, brains from 2.5 to 3 months old mice were dissected, their hippocampi removed and immediately placed in ice-cold homogenization buffer (10 mM Sucrose, 10 mM HEPES, supplemented with Protease Inhibitor Cocktail tablets (Roche, Burgess Hill, UK), pH 7.4). Tissue samples were homogenized with a teflon/glass homogenizer then centrifuged (1,000g, 10 min, 4°C). The supernatant was collected and centrifuged at 12,000g (20 min, 4°C), after which the pellet was resuspended twice in 4mM HEPES (containing 1mM EDTA, pH 7.4) by repeating the last centrifugation step. To obtain the non-PSD enriched fraction, pellets were then resuspended in 20 mM HEPES, 100 mM NaCl and 0.5% Triton X-100 (pH 7.2). Samples were incubated 15 min at 4°C whilst rotating gently, followed by centrifugation (12,000g, 20 min, 4°C). The supernatant was collected (Non-PSD enriched fraction) and the pellet solubilized (in 20 mM HEPES, 0.15 mM NaCl, 1% Triton X-100, 1% sodium deoxycholate (DOC), 1% SDS, 1 mM DTT, pH 7.5) for a further 1h at 4°C with gentle agitation. Finally, the samples were centrifuged at 10,000g for 15 min (4°C) and the supernatants were collected as PSD-enriched fractions. Fractions were stored at -20°C until Western Blotting, and used less than a week after preparation. Immunofluorescence was performed as described (McKenzie et al., 2005). Primary antibodies used: Anti-phospho (serine-133) CREB (1:100; Upstate Biotechnology, Lake Placid, NY, USA). Antibody binding was visualized using biotinylated secondary antibody/cy3-conjugated streptavidin, and nuclei were counter-stained with DAPI. Non-saturating pictures were taken on a Leica AF6000 LX imaging system, with a DFC350 FX digital camera and fluorescence intensity quantified on Image J (Wayne Rasband, U.S. National Institutes of Health, Bethesda, MD, USA). Average cellular fluorescence intensity was quantified for each picture taken, and all settings kept the same for pictures taken of other conditions/genotypes withing that experiment. For each condition/timepoint, 100 cells were analysed per replicate. For western blotting- in order to minimize the chance of post-translational modifications during the harvesting process, neurons were lysed immediately after stimulation in 1.5x sample buffer (1.5M Tris pH 6.8; Glycerol 15%; SDS 3%; β-mercaptoethanol 7.5%; bromophenol blue 0.0375%) and boiled at 100°C for 5 min. Approximately 30 µg of protein was loaded onto a precast gradient gel (4-20%) and subjected to electrophoresis. Western blotting onto a PVDF membrane was then performed using the Xcell Surelock system (Invitrogen) according to the manufacturer's instructions. Following the protein transfer, the PVDF membranes were blocked for 1 hour at room temperature with 5% (w/v) non-fat dried milk in TBS with 0.1% Tween 20. The membranes were incubated at 4°C overnight with the primary antibodies diluted in blocking solution: Anti phospho-CREB ser133 (1:500, Upstate), Anti-CREB (1:500, Cell Signaling, Danvers, MA, USA), anti-PSD-95 (1:1000, BD Biosciences, Oxford, UK), anti-GluN2B (N-terminus, 1:1500, Invitrogen), anti-nNOS (BD Biosciences). For visualisation of Western blots, HRP-based secondary antibodies were used followed by chemiluminescent detection on Kodak X-Omat film. Western blots were digitally scanned and densitometric analysis was performed using Image J. All analysis involved normalizing to either CREB (for phospho-CREB) or Akt expression as a loading control.

Co-immunoprecipitation

All steps were carried out at 4ºC. P7 cortices were homogenized in IP buffer (50 mM Tris HCl pH 9, 50 mM NaF, 20 µM $ZnCl_2$, 1 mM Na_3VO_4, 1:100 protease inhibitor cocktail set III (Merck Chemicals, Nottingham, UK), 0.5 mg/ml PMSF, 1% DOC) and centrifuged for 40 min at 25000g. At P7, 1% DOC is sufficient to solubilize the overwhelming majority of GluN2B (Al-Hallaq et al., 2007) and we have confirmed that >95% of GluN2B is solubilized with our IP buffer (data not shown). The protein concentration in the supernatant was measured (by BCA assay) and adjusted to 2 mg/ml using IP buffer. Antigen binding was carried-out by adding of 1 µg of antibody (anti-

GluN2B (N-terminus, Santa Cruz), or control IgG) to 1 mg of protein extract. The antibody-antigen mixture was incubated overnight with rotation at 4°C. 50 µl Dynabeads® were used per immunoprecipitation reaction incubation, and were aliquoted to each antigen-antibody mixture. Beads were incubated with the antibody for 20 min at room temperature on a rotator. The Dynabead-antibody-antigen complex was then washed four times in IP buffer, before being eluted by suspending the beads in 50 µl of 2x Laemmli buffer (with 5% 2-mercaptoethanol, Bio-Rad Life Sciences, Hemel Hempstead, UK) and boiling for 5 min. The supernatant was then stored at -80°C until SDS-PAGE and western blotting was carried out. For co-immunoprecipitation studies in HEK293 cells, cells were transfected with plasmids encoding GluN2B (and mutants thereof), GluN1, and PSD-95 in the presence of MK-801 to minimize HEK cell excitotoxicity. Protein was harvested for co-immunoprecipitation studies at 24 h post-transfection as per the protocol above.

Statistical analysis, equipment and settings.

Statistical testing involved a 2-tailed paired student T-test. For studies employing multiple testing, we used a one-way ANOVA followed by Fisher's LSD post-hoc test. For western blots, we used chemiluminescent detection on Kodak X-Omat film. Appropriate exposures were taken such that bands were not saturated. For figure preparation of example western blots, linear adjustment of brightness/contrast was applied (Photoshop) equally across the entire image, taking care to maintain some background intensity. In some cases, lanes from non-adjacent lanes are spliced together, but the lanes are always from the same blot, processed in exactly the same way, and the splicing point is clearly marked by a dark grey line.

Pictures of transfected neurons were taken on a Leica AF6000 LX imaging system, with a DFC350 FX digital camera. The DFC350 FX digital camera is a monochrome camera, and so coloured images (e.g. of green fluorescent protein) essentially involve taking a black and white image (using the appropriate filter set) and applying a colour to the image after capture. All luminescent assays were performed on a FLUOstar OPTIMA (BMG Labtech, Aylesbury, UK). Light collection time and gain were set such that counts were substantially lower than the maximum level collectable. All chemicals were obtained from Sigma Aldrich (Gillingham, UK) unless otherwise stated.

Supplemental References

Al-Hallaq, R.A., Conrads, T.P., Veenstra, T.D., and Wenthold, R.J. (2007). NMDA di-heteromeric receptor populations and associated proteins in rat hippocampus. J Neurosci *27*, 8334-8343.

Hardingham, G.E., Chawla, S., Johnson, C.M., and Bading, H. (1997). Distinct functions of nuclear and cytoplasmic calcium in the control of gene expression. Nature *385*, 260-265.

McKenzie, G.J., Stevenson, P., Ward, G., Papadia, S., Bading, H., Chawla, S., Privalsky, M., and Hardingham, G.E. (2005). Nuclear Ca2+ and CaM kinase IV specify hormonal- and Notch-responsiveness. J Neurochem *93*, 171-185.

Milnerwood, A., Gladding, C., Pouladi, M., Kaufman, A., Hines, R., Boyd, J., Ko, R., Vasuta, O., Graham, R., Hayden, M., *et al.* (2010). Early increase in extrasynaptic NMDA receptor signalling and expression contributes to phenotype onset in Huntington's disease mice. Neuron *65*, 178-190.

Minta, A., Kao, J., and Tsien, R. (1989). Fluorescent indicators for cytosolic calcium based on rhodamine and fluorescein chromophores. Journal of Biological Chemistry *264*, 8171-8178.

Rutter, A.R., and Stephenson, F.A. (2000). Coexpression of postsynaptic density-95 protein with NMDA receptors results in enhanced receptor expression together with a decreased sensitivity to L-glutamate. J Neurochem *75*, 2501-2510.

Soriano, F.X., Martel, M.A., Papadia, S., Vaslin, A., Baxter, P., Rickman, C., Forder, J., Tymianski, M., Duncan, R., Aarts, M., *et al.* (2008). Specific targeting of pro-death NMDA receptor signals with differing reliance on the NR2B PDZ ligand. J Neurosci *28*, 10696-10710.

Stehle, J.H., Foulkes, N.S., Molina, C.A., Simonneaux, V., Pevet, P., and Sassone-Corsi, P. (1993). Adrenergic signals direct rhythmic expression of transcriptional repressor CREM in the pineal gland. Nature *365*, 314-320.
